# Human DNA-Damage-Inducible 2 Protein Is Structurally and Functionally Distinct from Its Yeast Ortholog

**DOI:** 10.1038/srep30443

**Published:** 2016-07-27

**Authors:** Monika Sivá, Michal Svoboda, Václav Veverka, Jean-François Trempe, Kay Hofmann, Milan Kožíšek, Rozálie Hexnerová, František Sedlák, Jan Belza, Jiří Brynda, Pavel Šácha, Martin Hubálek, Jana Starková, Iva Flaisigová, Jan Konvalinka, Klára Grantz Šašková

**Affiliations:** 1 Gilead Sciences and IOCB Research Center, Institute of Organic Chemistry and Biochemistry of the Academy of Sciences of the Czech Republic, Flemingovo n. 2, 166 10 Prague 6, Czech Republic; 2 First Faculty of Medicine, Charles University in Prague, Katerinska 32, 121 08, Prague 2, Czech Republic; 3 Department of Biochemistry, Faculty of Science, Charles University, Hlavova 8, 128 00 Prague 2, Czech Republic; 4 Department of Physical and Macromolecular Chemistry, Faculty of Science, Charles University, Hlavova 8, 128 00 Prague 2, Czech Republic; 5 Groupe de Recherche Axé sur la Structure des Protéines, Department of Pharmacology & Therapeutics, McGill University, Montreal, QC, H3G 1Y6, Canada; 6 Institute for Genetics, University of Cologne, Zülpicher Str. 47a, 50647 Cologne, Germany

## Abstract

Although Ddi1-like proteins are conserved among eukaryotes, their biological functions remain poorly characterized. Yeast Ddi1 has been implicated in cell cycle regulation, DNA-damage response, and exocytosis. By virtue of its ubiquitin-like (UBL) and ubiquitin-associated (UBA) domains, it has been proposed to serve as a proteasomal shuttle factor. All Ddi1-like family members also contain a highly conserved retroviral protease-like (RVP) domain with unknown substrate specificity. While the structure and biological function of yeast Ddi1 have been investigated, no such analysis is available for the human homologs. To address this, we solved the 3D structures of the human Ddi2 UBL and RVP domains and identified a new helical domain that extends on either side of the RVP dimer. While Ddi1-like proteins from all vertebrates lack a UBA domain, we identify a novel ubiquitin-interacting motif (UIM) located at the C-terminus of the protein. The UIM showed a weak yet specific affinity towards ubiquitin, as did the Ddi2 UBL domain. However, the full-length Ddi2 protein is unable to bind to di-ubiquitin chains. While proteomic analysis revealed no activity, implying that the protease requires other factors for activation, our structural characterization of all domains of human Ddi2 sets the stage for further characterization.

The ubiquitin-proteasome system (UPS) plays a crucial role in eukaryotic cell biology. Pathway components are involved in processes including protein degradation and trafficking, cell signaling, response to DNA damage, and cell cycle regulation. Ubiquitin (UBQ) is a central molecule in the pathway, and its ability to form various polymeric chains marks substrates for specific tasks^1^,^2^. Controlling mechanisms by which the chains are recognized are important for proper system function and cellular homeostasis. Imbalance in any step of the pathway can have significant impact on an organism, and thus, complete understanding of this central pathway is essential.

Polyubiquitination marks proteins for multiple fates, such as degradation or vesicle sorting. Polyubiquitinated proteins that undergo degradation are either recognized directly by proteasomal receptors (Rpn10, Rpn13) or “captured” by so-called shuttle (or adaptor) proteins (Rad23, Dsk2, and Ddi1 in budding yeast). The shuttles deliver their polyubiquitinated substrates to the regulatory part of the 26S proteasome^3^,^4^,^5^,^6^,^7^,^8^,^9^. Proteasomal shuttle proteins possess a typical domain architecture that includes an N-terminal ubiquitin-like domain (UBL) that binds the 26S proteasome and a C-terminal ubiquitin-associated domain (UBA) responsible for binding UBQ or poly-UBQ chains^10^.

In line with this UBL-UBA domain architecture, DNA damage-inducible (Ddi1)-like proteins are thought to act as proteasomal shuttle proteins, although the evidence for this function is incomplete^9^,^10^,^11^,^12^. Recently, Nowicka and co-workers proposed an alternative mechanism for the yeast Ddi1 (yDdi1) shuttling process based on the surprising fact that yDdi1 UBL binds UBQ^13^. Yet another factor differentiates Ddi1-like proteins from classical proteasomal shuttles: Ddi1-like proteins contain an additional domain called the retroviral protease-like (RVP) domain, the 3D fold of which is strikingly reminiscent of HIV-1 protease. RVP is highly conserved in eukaryotes, and is present in human Ddi1-like orthologs. It contains the catalytic triad characteristic of aspartic proteases (D[T/S]G) and is responsible for dimerization of the protein (Fig. 1A)^11^,^14^. The physiological substrate of this putative aspartic protease, if any, remains unknown.

Ddi1 from *Saccharomyces cerevisiae* is by far the best-studied Ddi1-like ortholog. Its expression is DNA-damage inducible, and it is involved in cell cycle progression through the mitotic checkpoint protein Pds1^15^,^16^. Studies from the Raveh laboratory indicate that it plays a role in degradation of HO endonuclease, the enzyme responsible for switching alleles at the mating type locus *MAT*^9^. Furthermore, yDdi1 interacts with the exo- and endocytotic v-SNARE proteins Snc1 and Snc2 as well as exocytotic t-SNARE Sso1, playing a role as a negative regulator of exocytosis^11^,^17^,^18^.

Overall, the current body of knowledge indicates that Ddi1-like proteins play a significant role in cell cycle control, growth control, and trafficking in yeast and may play a crucial role in embryogenesis in higher eukaryotes. Ddi1-like orthologs from higher eukaryotes have not been investigated in much detail. Notably, Ddi1-like protein from *Caenorhabditis elegans* (Vsm-1) may play a crucial role in synaptogenesis^19^. In *Drosophila melanogaster*, knock-out of the *Rngo* (fruit fly *DDI1* homolog) gene is lethal and forms ring canal defects in oogenesis^20^. Moreover, a high-throughput proteomics study identified Rngo protein as one of the most abundant ubiquitinated proteins during neural development in *Drosophila* embryogenesis^21^.

The highly conserved RVP domain poses an interesting evolutionary puzzle. The 3D structure of yDdi1 RVP was solved by others (PDB code 2I1A)^22^ at 2.3 Å resolution and very recently by us at 1.9 Å resolution. Our structure shows the conformation of the “flap” region in detail (HIV terminology), which was missing in the previous model (details are presented in our back-to-back publication, Trempe *et al.*, 2016)^22^,^23^,^24^. However, the structure of the RVP domain of human Ddi2 (hDdi2) has not been published to date. The putative active site of yDdi1 RVP is similar to that of HIV-1 protease, including a water molecule that could act as a nucleophile for peptide bond hydrolysis. The first direct evidence that Ddi1-like RVP can act as a protease was presented by Perteguer and coworkers, who showed that a *Leishmania major* Ddi1-like ortholog cleaves BSA at acidic pH^25^. In addition, they showed that it hydrolyzes one HIV peptide substrate and two cathepsin D substrates and that this activity can be inhibited by specific aspartic protease inhibitors. This evidence was supported by another finding showing that knock-out of yDdi1 leads to an increase in protein secretion into the media^17^ and can be complemented by transfection of a plasmid encoding Ddi1. Complementation requires both the UBL and Asp220 of the RVP active site^26^. White and coworkers reported the similar finding that the yDdi1 knock-out phenotype can be rescued by a plasmid encoding human or leishmanial Ddi1. This rescue is inhibited by some HIV protease inhibitors^27^. Data obtained with Rngo, the Ddi1-like ortholog from *Drosophila*, also supports the hypothesis that Ddi1 is an active protease: the oogenesis-defect phenotype can be fully rescued by transgenes encoding full-length Rngo or Rngo lacking either the UBL or UBA domain. In contrast, the phenotype cannot be rescued by Rngo protein variant with a mutated catalytic aspartate in the RVP domain (D257A)^20^. Therefore, it is clear that Ddi1-like RVP is required for its biological function, although its physiological substrate remains elusive.

In the human genome, there are two genes (located on chromosome 11 and chromosome 1) encoding Ddi1-like proteins: the 396-amino-acid Ddi homolog 1 (hDdi1) and the 399-amino-acid Ddi homolog 2 (hDdi2). Based on its genomic organization, hDdi2 seems to be the “original” version of yDdi1 that later gave rise to hDdi1 through a retrotransposition event. To the best of our knowledge, neither protein has been specifically studied. They share 70% amino acid sequence identity and 81% similarity. Compared to the protein domain architecture of lower eukaryotes that of both human variants is conserved only to a certain extent. While the UBL and RVP domains are preserved, the UBA domain is missing. Therefore, the putative function of human Ddi1-like proteins as proteasomal shuttles is questionable, and their biological role remains elusive.

We present here the first structural and functional study of hDdi2. We first analyze the evolutionary pathway leading to the loss of the UBA domain. We identify a putative short UBQ-interacting motif (UIM) at the C-terminus, instead of UBA, and we show its specific but very weak binding to UBQ. Prompted by the recent results from Nowicka and coworkers, we solved the 3D structure of hDdi2 UBL and performed NMR titrations with UBQ. While the yDdi1 UBL binds to UBQ^13^,^23^, we observe only a weak affinity of hDdi2 UBL for UBQ. We extended our investigations to UBQ conjugates and showed that hDdi2 does not bind any di-UBQ chains *in vitro*. We also present the first 3D structure of the hDdi2 RVP domain, together with its functional proteolytic analysis. Finally, we used NMR to elucidate the structure of the region preceding the RVP domain, which we named the Helical Domain of hDdi2 (HDD), and describe its characteristic features.

## Results

### Evolution of Ddi1-like proteins: loss of UBA and identification of a novel ubiquitin-interacting motif in human Ddi2

Ddi1-like proteins, which combine an N-terminal UBL domain with an intact RVP, arose early in eukaryotic evolution. Database searches with sequence profiles for UBL and RVP domains have detected widespread occurrence of these proteins in animals, plants, and fungi^28^, as well as in protozoan lineages including apicomplexans, kinetoplastids, and oomycetes. The majority of UBL-RVP containing proteins also possess a C-terminal UBA domain, suggesting that they might act as proteasomal shuttling factors similar to yDdi1^29^. However, Ddi1-like proteins from all vertebrate families appear to have lost the UBA domain, although it is retained in other animal lineages. In the mammalian lineage, the UBA-deficient gene was duplicated, giving rise to two related UBL-RVP-containing genes, called *DDI1* and *DDI2* in humans. Despite their names, yDdi1 and its non-mammalian homologs are more similar to hDdi2 than to hDdi1. Because the human *DDI2* gene also shares conserved synteny with the single *DDI1*-like gene of non-mammalian vertebrates, *DDI2* is assumed to be the “original” version that later gave rise to the intron-less mammalian *DDI1* through a retrotransposition event.

Closer inspection of the mammalian *DDI2* locus and corresponding loci in non-mammalian vertebrates shed light on the evolutionary fate of the C-terminal UBA domain. Early in vertebrate evolution, a novel vertebrate-specific gene called *RSC1A1* apparently became inserted into the ancestral *DDI2* locus, separating the N-terminal UBL-RVP portion from the C-terminal UBA-containing region. In extant vertebrates, the UBA domain has become part of the RSC1A1 polypeptide and might participate in this protein’s function of regulating the trafficking of sugar transporters^30^.

**Figure 1:**
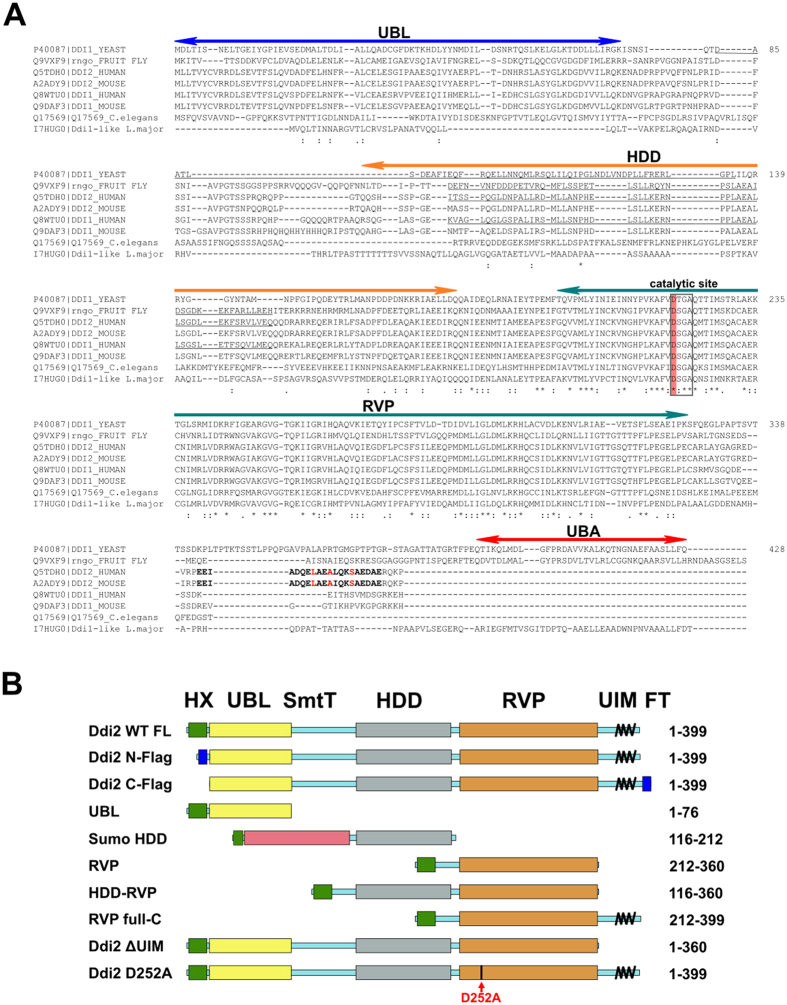
Sequence analysis of Ddi1 orthologs. (**A**) Sequence alignment of Ddi1-like proteins from various eukaryotic organisms. Domains are indicated with double-headed arrows. The highly conserved catalytic site of RVP is highlighted. The putative UIM motif is highlighted in bold, with residues important for ubiquitin binding in red. (**B**) Schematic diagram of full-length hDdi2 and the truncated constructs used in this study. Positions of the histidine tag including the factor Xa cleavage site (green), UBL domain (yellow), HDD (gray), RVP domain (orange), and C-terminal UIM (black helix) are indicated. Flexible regions are indicated with blue boxes. Mutation of the putative catalytic aspartate (D252A) is indicated with a red arrow.

Considering the putative role of hDdi2 as a shuttle protein for the UPS, we performed a bioinformatics analysis of the newly evolved C-terminus to identify potential alternative UBQ-binding domains to the lost UBA domain. Alignment of Ddi1-like sequences from various organisms revealed a conserved region of 24 residues that is absent from yDdi1 and non-vertebrate Ddi1-like sequences. Comparison of this region to databases of annotated domains using the program HHPRED revealed significant similarity (p < 0.0001) to a family of ubiquitin-interacting motif (UIM) proteins^31^. As shown in Fig. 1, the pattern of UBQ-binding residues typical of UIM motifs is conserved in the Ddi2 family, suggesting that this newly identified UIM-like motif might replace the lost UBA domain as a UBQ receptor.

### The C-terminal UIM motif of human Ddi2 binds weakly, yet selectively to mono-UBQ

**Figure 2:**
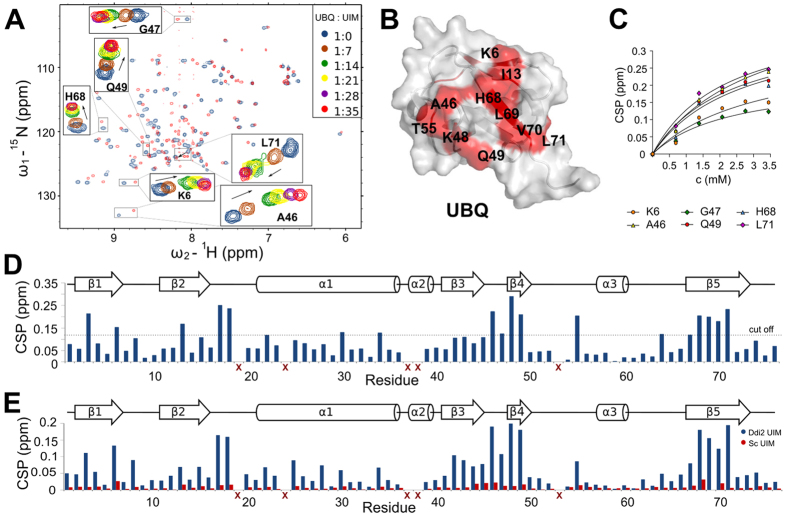
Mapping of the UBQ-hDdi2 interaction site. (**A**) ^15^N/^1^H-HSQC titration spectra of UBQ with hDdi2-UIM peptide. (**B**) Identification of mapped residues shown on the UBQ structure (PDB entry 1D3Z)^32^. (**C**) Titration curves of selected amino acids on UBQ. (**D**) Plot of chemical shift perturbations of individual amino acids upon interaction at the end point of the titration (35-fold molar excess). Red crosses mark amino acids that were not reliably observed in the titration spectra. (**E**) Plots of chemical shift perturbations of UBQ residues upon interaction with 2.2 mM hDdi2-UIM peptide (blue) and upon addition of hDdi2-scrambled UIM peptide (red) to a final concentration of 1.9 mM.

To evaluate the putative ability of the C-terminal UIM of hDdi2 to bind UBQ, we performed NMR chemical shift perturbation (CSP) experiments with UBQ and either 1) hDdi2-UIM peptide (hDdi2 residues 376–396); 2) hDdi2-scrambled UIM peptide; 3) the full C-terminus of Ddi2 including the RVP domain (hDdi2 RVP-UIM full-C, residues 212–399). After assignment of both double and triple resonance spectra of ^15^N and ^15^N/^13^C-labeled protein constructs (RVP full-C and UBQ), we analyzed specific shifts in positions of backbone amide signals induced by the addition of non-labeled peptide or protein partner (Fig. 2).

First, we titrated UBQ with UIM peptide. We reached a UIM peptide concentration of 3.45 mM (35-fold molar excess over UBQ) and determined the K_d_ between 2.2–3.2 mM. The K_d_ was calculated from 6 residues (Lys6, Ala46, Gly47, Gln49, His68, and Leu71) by fitting the titration curves with a 1:1 stoichiometry model for specific binding (Fig. 2C). The CSPs are illustrated in the overlaid spectra, with and without final addition of the peptide, with a close-up on significantly shifted peaks (used for K_d_ calculation) that were mapped onto the UBQ structure (PDB 1D3Z) (Fig. 2A,B)^32^. Based on shifts in residues used for fitting the titration and in Leu8, Arg42, Lys48, Gln49, and Leu71, we concluded that the binding epitope is slightly different compared to the Ile44 hydrophobic patch (Fig. 2D). However, we observed different shifts in backbone amides of other amino acids (Ile3, Ile13, Val17, Glu18, Glu34, Thr55, Glu64, and Leu69). The control experiment with the hDdi2-scrambled UIM peptide revealed no significant CSPs in comparison to equimolar addition of the hDdi2-UIM peptide (Fig. 2E), suggesting that the weak interaction between the UIM and ubiquitin is nonetheless specific.

Guided by previous NMR data with isolated motifs, we next examined binding of ^15^N-labeled UBQ with addition of a 1-, 2-, and 5-fold molar excess of non-labeled hDdi2 RVP full-C, which could provide a more refined map of the interaction (Figure S1A). Relatively small yet specific changes in positions of backbone signals were observed for residues Thr7, Arg42, Lys48, Gln49, and Leu71, which were slightly different from those seen in the Ile44 patch known to interact with several UBAs and UIM^10^,^24^,^33^ (Figure S1A). We also performed the reverse experiment with ^15^N-labeled hDdi2 RVP full-C protein and addition of a 1-, 2-, and 5-fold molar excess of non-labeled UBQ. The alignment of HSQC spectra during the titration revealed shifts in individual residues located at the Ddi2-UIM peptide sequence (Figure S1B). Overall, the data suggest that UBQ binds to the C-terminal sequence harboring the putative UIM, but with very weak affinity.

Inspired by the work of Singh and co-workers showing specific interaction of yDdi1 and Rub1 (the closest relative of UBQ, Nedd8 in mammals)^34^, we performed similar NMR CSP experiments to investigate the possibility of Nedd8 binding to hDdi2. In this case, we did not observe any significant perturbation with the C-terminal hDdi2 UIM peptide (Figure S2) nor with the N-terminal UBL domain of hDdi2 (Figure S3A). Therefore, we conclude that the C-terminal UIM of hDdi2 specifically binds UBQ.

### The UBL domain from human Ddi2 binds more weakly to ubiquitin than the yeast Ddi1 UBL

**Figure 3:**
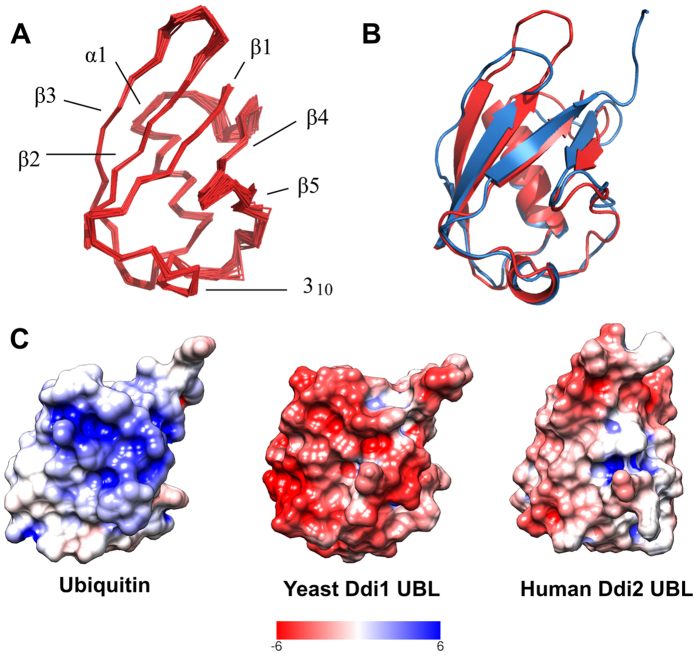
Solution structure of the hDdi2 UBL domain. (**A**) Superimposition of 40 converged structures of the UBL domain. (**B**) Structural alignment of solution structures of the yDdi1 UBL in blue (PDB code 2N7E) and hDdi2 UBL in orange (PDB code 2N7D). The structural alignment over 74 equivalent positions yields an RMSD of 1.66 Å^36^. (**C**) Comparison of the surface electrostatic potential of ubiquitin (PDB 1UBQ), yDdi1 UBL (accompanying paper by Trempe)^23^, and hDdi2 UBL. For NMR structures, representative structures closest to the mean structure were used, but similar results were obtained with the first structures of the ensembles. All molecules are oriented based on secondary structure alignment, with the β-sheet area towards the reader. The surface is colored from red (negative values) to blue (positive values); the range is ±6 kT/e for all structures. Surface electrostatic potential maps were generated using the Adaptive Poisson Boltzmann Solver^57^ package with structure preprocessing using the PDB2PQR tool^58^ in the UCSF Chimera software package^55^. All calculations were performed using the SWANSON force field at pH 7.4; other settings were kept at default values. Chimera was also used for final surface visualization.

To gain deeper structural information about hDdi2, we obtained nearly complete ^15^N-, ^13^C-, and ^1^H-resonance assignments of its N-terminal UBL domain (residues 1–76, with N-terminal histidine tag) and determined the solution structure with high precision. The root mean-squared deviation (RMSD) to the mean structure for the backbone and heavy atoms for the final 40 converged structures was 0.4 Å overall and 1 Å at the ordered residue range (residues 1–76 of the protein sequence). The UBL of hDdi2 contains five β-sheets (β1: M1-V8, β2: V15-V21, β3: Q46-Y49, β4: R52-P53, β5: V71-R75), one α-helix (L27-S38), and a 3_10_-helix (L61-Y64), which is consistent with the typical UBQ β-grasp fold (Fig. 3A). The distribution of NMR constraints and structural statistics for the hDdi2 UBL domain are summarized in Table S1.

To characterize the binding properties of hDdi2 UBL, we inspected its structure and performed a detailed comparison with the UBL structure of yDdi1 reported in our back-to-back publication^23^. The sequence similarity between the yeast and human UBL domains is 46%, and despite their low sequence identity (25%)^35^, their secondary structure elements superimpose very well with a backbone RMSD of 1.66 Å^36^ (Fig. 3B). We compared the surface properties of the interaction patches from both yDdi1 and hDdi2 UBLs and UBQ (Fig. 3C). As discussed by Nowicka and co-workers^13^, the β-sheet interaction area of yDdi1 UBL is formed by positively charged side chains, which makes it complementary to the negatively charged UBQ patch. Interestingly, the surface electrostatic potential of hDdi2 UBL shows a small hydrophobic area that is moderately charged. We reasoned, that due to different charge distribution on the interaction patch of hDdi2 UBL and yDdi1 UBL, they might interact with different partners.

**Figure 4:**
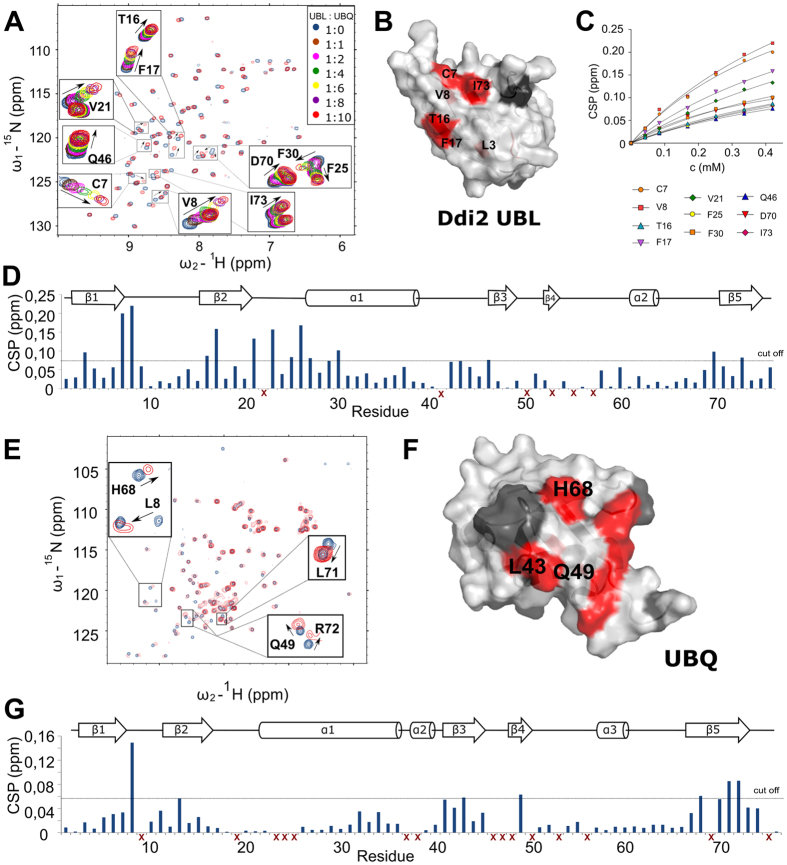
Characterization of the hDdi2 UBL interaction with UBQ. (**A**) ^15^N/^1^H-HSQC titration spectra of Ddi2 UBL with addition of a 1-, 2-, 4-, 6-, 8-, or 10-fold molar excess of UBQ. Residues Cys7, Val8, Thr16, Phe17, Val21, Phe25, Phe30, Gln46, Asp70, and Ile73 were used for K_d_ calculation (0.42–1.1 mM). (**B**) The mapped interaction site shown on the UBL structure is most likely located in the β-sheet area, according to shifts in Leu3, Cys7, Val8, Thr16, Phe17, and Ile73 upon UBQ binding. Additional shifts in backbone amides observed in the spectra (Val21, Ala23, Phe25, Glu26, Phe30, and Asp70) at the other site of the domain could be the result of a structural change upon binding. Amino acids that could not be used for evaluation are marked black. (**C**) Titration curves of selected hDdi2 UBL amino acids used for K_d_ calculation according to the 1:1 stoichiometry model for specific binding. (**D**) CSP plot showing perturbation at the titration endpoint. Residues not considered in the evaluation are marked with red crosses. (**E**) ^15^N/^1^H-HSQC titration spectra of UBQ with final 6-fold excess of hDdi2 ΔUIM with close-ups of the shifted signals of individual amino acids mapped (**F**) onto UBQ (PDB entry 1D3Z) (**G**) Plots of chemical shift perturbations of individual amino acids of UBQ.

Prompted by the unexpected finding of Nowicka and co-workers that yDdi1 UBL binds UBQ with a K_d_ of 45 ± 7 μM, we investigated whether hDdi2 UBL has any affinity for UBQ^13^. We performed NMR titration experiments on ^15^N-labeled hDdi2 UBL with addition of UBQ up to a 10-fold molar excess (Fig. 4A). We mapped the most relevant shifts onto the structure of hDdi2 UBL (Fig. 4B), which showed that this interaction is located in the β-sheet area, with a K_d_ in the 0.42–1.1 mM range, calculated from 10 residues (Fig. 4C). This interaction was supported by a reverse experiment with ^15^N-labeled UBQ titrated with non-labeled hDdi2 ΔUIM (lacking UIM) to a 6-fold molar excess. We mapped the changes in HSQC spectra onto the site close to Ile44 patch (Fig. 4E–G). A negative control experiment with 6-fold molar addition of hDdi2 HDD-RVP (lacking both UIM and UBL) did not show any significant CSPs of the UBQ backbone amide signals (Figure S3B). On the basis of these data, we infer that unlike the yDdi1 UBL domain, the hDdi2 UBL domain interacts weakly with UBQ with a Kd in the low millimolar range.

We next examined whether the UBL of hDdi2 could bind the protein’s C-terminal UIM motif. We performed NMR titration experiments with ^15^N-labeled hDdi2 UBL with addition of hDdi2-UIM peptide to a final concentration of 1.9 mM (Figure S3C), as well as negative control experiment with the same molar addition of hDdi2-scrambled UIM peptide. Both resulted in the same low CSP response (Figure S3C). We next measured and superimposed HSQC spectra of ^15^N-labeled full-length hDdi2 and the ΔUIM truncated form of hDdi2 to elucidate the potential intramolecular interaction (Figure S3D). No difference was observed in the chemical shifts corresponding to the hDdi2 UBL domain, suggesting that hDdi2 UBL cannot bind its own C-terminal UIM and most likely never adopts a “head-to-tail” auto-inhibited conformation. Interestingly, superimposition of the HSQC spectra of ^15^N-labeled full-length protein with its UBL domain revealed shifts in almost all N-terminal amino acids of hDdi2 (Figure S3E). This demonstrates that the UBL domain binds and is not independent from the rest of the protein, in contrast to the yDdi1 UBL^13^,^23^.

### Polyubiquitin chain binding is not preserved in human Ddi2

**Figure 5:**
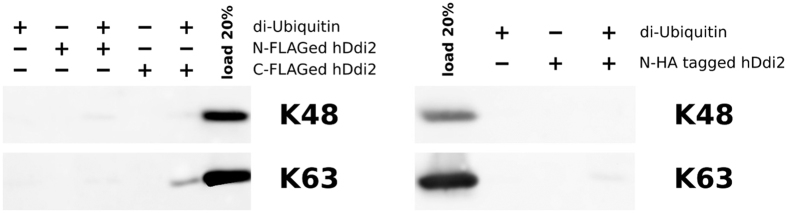
Human Ddi2 shows no strong interaction with di-ubiquitin chains. Western blot analysis of pull-down experiments with di-ubiquitin conjugates of Lys48 and Lys63 architecture. Human Ddi2 with a FLAG tag on either the N- or C-terminus or an HA tag on the N-terminus was immobilized on magnetic agarose beads. Beads were incubated with the di-ubiquitin conjugate of given linkage architecture, washed, and eluted by boiling in non-reducing SDS sample buffer. Samples were analyzed on 18% SDS-PAGE followed by immunoblotting with anti-UBQ antibody.

Given that the interaction between hDdi2 and mono-UBQ is very weak and completely different from that of yDdi1 and UBQ, we wondered whether these weak interactions mediated by the UBL and UIM motifs could synergize to enable polyvalent binding to ubiquitin chains. Therefore, we tested the binding full-length hDdi2 to various UBQ chains (Fig. 5, Figure S4). N- and C-terminally FLAG-tagged hDdi2 and HA-tagged hDdi2 were immobilized on magnetic beads and mixed independently with all eight native linkage types of di-UBQ conjugates (Lys6-, Lys11-, Lys27-, Lys29, Lys33-, Lys48-, Lys63-linked, and linear). The same experiment was repeated also with in house synthetized Lys48- and Lys63-linked chains. The data clearly shows that hDdi2 does not pull down any of di-UBQ conjugates under physiological pH. This contrasts with yDdi1, which binds to polyubiquitin chains^10^.

### The structure of the helical domain of human Ddi2 reveals a conserved bundle fold

Given the weak interaction of hDdi2 with ubiquitin, we looked for other domains in the protein to gain further insight into the function of the protein. Bioinformatics sequence analysis revealed strong conservation in the region preceding the RVP domain of hDdi2 (positions 116–212; Fig. 1). Within this region, we detected similarity to the Sti1 domain (residues 125–178), an α-helical domain found in the proteasome shuttle proteins Rad23 and Dsk2 and their animal homologs (Figure S5). The remainder of the region shows helicity as well, but does not share detectable similarity with other protein families. We refer to the entire α-helical bundle spanning residues 125–212 as the helical domain of Ddi (HDD).

**Figure 6:**
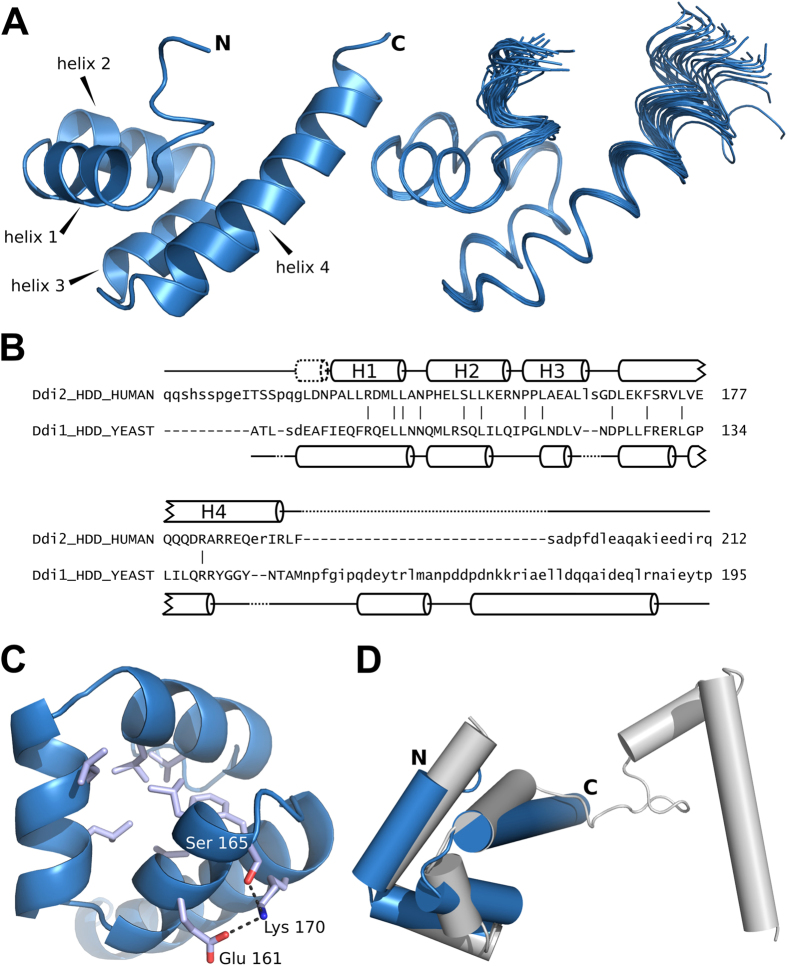
Solution structure of hDdi2 HDD. (**A**) Superimposition of 30 converged structures of HDD. (**B**) Structural alignment of hDdi2 HDD and yDdi1 HDD (PDB code 5KES) analyzed by Dali Pairwise comparison^37^. The Z score for these two structures is 4, and their RMSD is 5 Å. Secondary structures are shown; bars connect identical amino acids. (**C**) Hydrophobic core of the HDD bundle supported by a salt bridge between helix 3 and the initial part of helix 4, including residues Ser165 and Lys170, with occasional contribution from Glu161 (**D**) Superimposition of hDdi2 HDD (blue) with yDdi1 HDD (grey) represented by cylindrical helices. N-terminal parts of both HDDs superimpose with an RMSD of 0.95 Å.

The NMR structure of the hDdi2 HDD domain confirmed our prediction that this region adopts an α-helical folded structure (Fig. 6A and Table S1). The hDdi2 HDD structure consists of a globular arrangement of 4 α-helices spanning the following residues (Fig. 6B): helix 1 (135–144), helix 2 (146–155), helix 3 (157–164), and helix 4 (168–190). The region is preceded by two turns of another α-helix that is not included in the numbering. All four major helices pack against each other, forming a compact bundle with a hydrophobic core made up mostly of leucine residues. The bundle is further supported by a salt bridge between helix 3 and the initial part of helix 4, including residues Ser165 and Lys170, with occasional contribution of Glu161 (Fig. 6C). Helix 4 spans 22 amino acids with an interesting accumulation of 6 arginine residues in proximity to Arg153 from helix 2. The end of helix 4 is flexible. Both the N- and C-terminal parts of HDD form unstructured linker regions, allowing flexibility between the individual structured domains of hDdi2.

We used the Dali server^37^ to test whether HDD has structural homology with other known proteins, but surprisingly, we did not detect any significant structural homologs. We were also unable to manually superimpose the Sti1-like domain of Rad23 (PDB code 1 × 3W)^38^ with our HDD structure, although they show broad similarities. Next, we examined the structural homology between yDdi1 HDD and hDdi2 HDD, which share 25% sequence identity^39^. As shown in Fig. 6D, yDdi1 HDD forms two independent subdomains connected by a flexible linker^23^. Superimposition of the N-terminal “bundle” region of both HDDs (hDdi2 HDD residues 116–178, yDdi1 HDD residues 86–134) yielded an RMSD of 0.95 Å (Fig. 6D), whereas the RMSD calculation for the full-length structures expectedly yielded a high number (3.55 Å). This led us to hypothesize that the two-domain architecture of yeast HDD is in human HDD compacted into a single bundle with an extremely long final helix. We conclude that the hDdi2 HDD possesses a novel α-helical architecture.

### The human Ddi2 RVP domain adopts an aspartic protease-like structure

**Figure 7:**
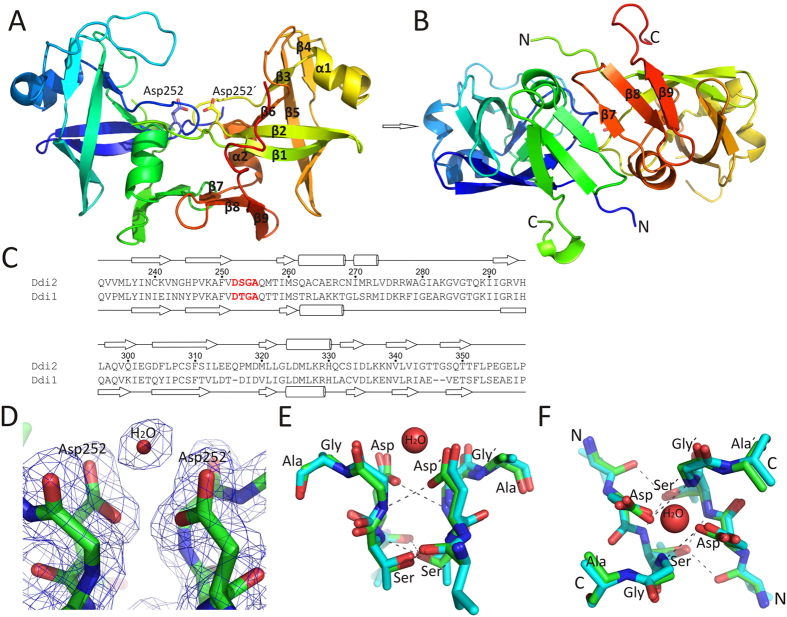
X-ray structure of the hDdi2 RVP domain. (**A**) A ribbon diagram of the structure of the hDdi2 RVP (residues 212–360) dimer (blue N-terminus to red C-terminus). The aspartate side chains that form the putative RVP active site are shown in stick representation. Secondary structure elements are labeled. (**B**) Second view of the RVP dimer related to A) by a 90° rotation about the horizontal axis. C- and N-termini, as well as secondary structure elements of the β-sheet platform, are highlighted. (**C**) Sequence alignment between the hDdi2 and yDdi1 RVP (PDB 2I1A)^22^ domains spanning residues from Gln232 to Pro359 of Ddi2, which are visible in the structure. Secondary structure elements are indicated, with arrows representing β-strands and cylinders representing α-helices of the hDdi2 RVP structure (above the sequence) and yDdi1 RVP (below). The putative active site of both RVP domains is highlighted in red. (**D**) The putative active site of the hDdi2 RVP domain showing catalytic aspartates and a water molecule, with the calculated omit map contoured at 1.0 σ. (**E**) The same section of the hDdi2 RVP (in green) shown in (**D**) superposed with the yDdi1 RVP domain^23^ (in blue). The hydrogen bonding pattern forming the “fireman’s grip” is indicated with dotted gray lines. (**F**) The same section shown in (**E**) rotated by 90° about the horizontal axis. C- and N-termini are indicated.

Next, we determined the crystal structure of the hDdi2 RVP domain (Ddi2 212–360) at 1.9 Å resolution (Fig. 7 and Table S2). The structure was solved by molecular replacement using PDB 2I1A as a starting model and refined to an *R*_*work*_*/R*_*free*_ of 20.8/21.6%^22^. Comparison of the hDdi2 RVP structure with the previously reported yDdi1 RVP structure revealed conservation of the overall fold (Fig. 7A,B) and active site (Fig. 7E,F)^22^. Similar to yDdi1 RVP, hDdi2 RVP comprises a six-stranded β-barrel, three β-sheet dimerization platform, and two helices, with the latter quite atypical for retroviral proteases. The second helix precedes the loop that corresponds to the flap region characteristic of other retroviral proteases. The flap in our hDdi2 RVP structure covers the active site only to a certain extent and cannot form hydrogen bonds with the second flap loop, unlike, for example, the structure of HIV-1 protease. The substrate cavity is thus significantly larger than those of other retroviral proteases and potentially could even accommodate small proteins, as observed previously in the yeast Ddi1 RVP^22^.

The putative catalytic cavity is formed by the typical amino acid signature of aspartic proteases (Asp-Ser-Gly-Ala). In yDdi1 RVP, Thr is present in place of Ser in the tetrapeptide. The RMSD for all atoms that form the Asp-Ser/Thr-Gly-Ala motif in the hDdi2 RVP and yDdi1 RVP structures is 0.353 Å. The RMSD calculated for the same monomer is 0.219 Å. Both values indicate perfect superposition of the active sites. Similar to other aspartic proteases, in hDdi2 RVP the putative catalytic Asp252 points to the area between the two β-barrel lobes. The residue following Asp252 is Ser, the side chain hydroxyl group of which participates in the “fireman’s grip” by hydrogen bonding to the backbone amide group of Ser253´ across the dimer interface and to the backbone carbonyl group of Val251´ (Fig. 7E,F). In agreement with structures of other aspartic proteases, we found a catalytic water molecule within hydrogen bonding distance of the Asp dyad. In summary, the geometry of the hDdi2 RVP domain structure corresponds to that of other catalytically active aspartic proteases, although the catalytic cavity seems to be more open and could possibly accommodate larger substrates.

### Small-angle X-ray scattering reveals that Ddi2 adopts an extended dimeric structure

**Figure 8:**
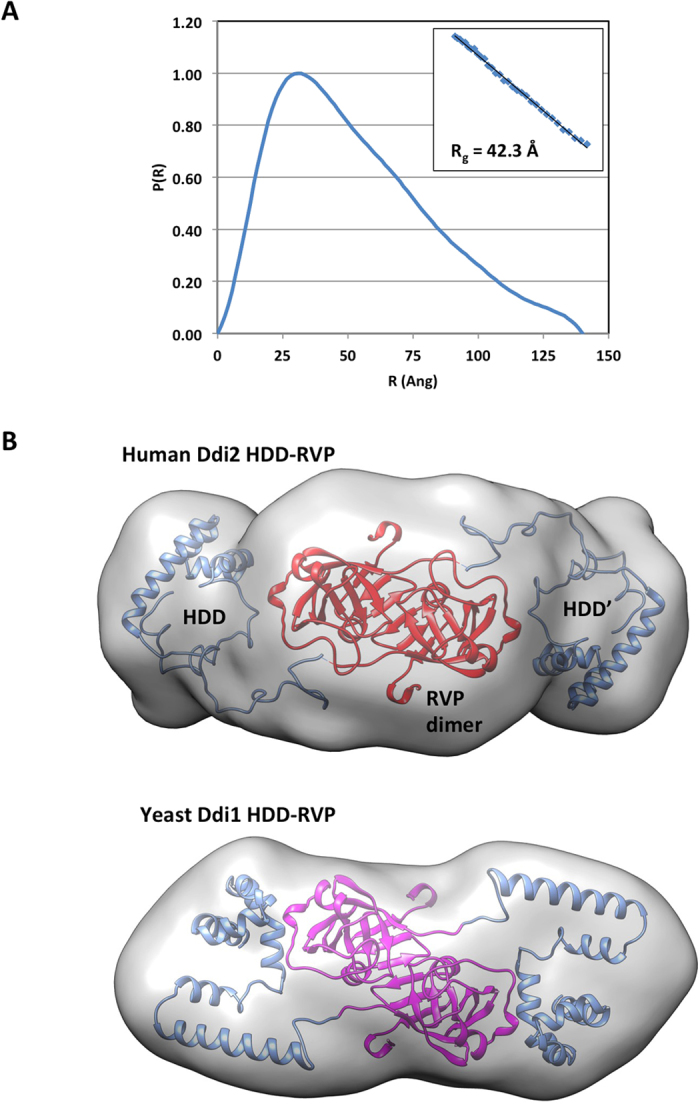
SAXS analysis of the HDD-RVP domains of hDdi2. (**A**) Pair-distance distribution from merged SAXS data, showing the asymmetric distribution characteristic of elongated structures. The inset shows the linearity of the Guinier plot for data collected at 5 mg/ml, indicating monodispersity. (**B**) Modeling of the HDD-RVP structure using the program BUNCH. Twenty models were superposed, averaged and converted to a map for surface visualization in Chimera (top). The structure of the HDD and RVP domains are displayed in blue and red, respectively for the two symmetry-related chains. The structure of the HDD-RVP module from yeast Ddi1 in showed at the bottom for comparison (back-to-back paper for details^23^).

To further inspect the overall shape of hDdi2, we used small-angle X-ray scattering (SAXS) to evaluate the molecular weight, radius of gyration, and low-resolution structure of the HDD-RVP domains of hDdi2. The SAXS invariant *V*_*c*_ was used to calculate a molecular mass of 66 kDa, which corresponds to the expected dimer mass (monomer: 30 kDa). The large *R*_*g*_ value of 42 Å and the *P(r)* distribution suggest an elongated structure (Fig. 8A). Modeling of the dimeric structure using the crystal structure of the RVP domain and NMR structure of HDD revealed that the HDD extends on either side of the RVP, similar to the yDdi1 HDD-RVP model with a slightly larger D_max_ of 140 Å (Fig. 7B). The overall larger dimensions of the hDdi2 HDD-RVP module arise from the longer flexible linker between the HDD N-terminal bundle and the RVP (40 residues), which in yeast Ddi1 is a more rigid two-helix segment connected by only 9 residues to the RVP. In hDdi2, the longer linker allows for the HDD bundle to extend further and adopt greater range conformations, which increases *D*_*max*_ and *R*_*g*_. Overall, the SAXS data confirmed the dimeric nature of hDdi2 in solution and the conserved structure of the HDD-RVP module between yeast and human Ddi1-like proteins.

### Search for putative proteolytic activity and small-molecule binder of the RVP domain

To shed light on the putative proteolytic activity of RVP, we performed PICS with full-length hDdi2 expressed in bacterial and mammalian expression systems^40^. In both cases, the cleavage experiment was performed with a mammalian-cell-derived peptide library prepared using trypsin and GluC digestion. We analyzed the cleavage profile of full-length hDdi2 at pH 4.0, 5.0, and 7.0 with 300 mM NaCl. As negative controls, we used hDdi2 with a D252A mutation in the putative catalytic site and a mock reaction with buffer instead of enzyme. As a positive control, we tested the HIV-1 protease cleavage profile in 100 mM Na acetate, 300 mM NaCl, pH 4.7, using wild-type enzyme and the catalytically inactive D25N mutant with a 1:200 protease-to-library ratio. To our surprise, the data analysis showed no cleavage related to hDdi2 (Figure S6).

Driven by this finding, we subjected the hDdi2 RVP domain to a similar enzymatic analysis as previously reported by Perteguer and co-workers, who showed BSA and HIV-peptide-derived substrate cleavage by leishmanial Ddi1 in acidic conditions^25^. We therefore tested BSA, HSA, β-casein, insulin, and a complete set of HIV-polyprotein-derived peptide substrates for putative hydrolysis by hDdi2 RVP at pH 5.0 and pH 7.0 in various salt concentrations (150 to 500 mM NaCl) by HPLC assay. Again, we did not observe any cleavage (Figures S7–14). ITC further demonstrated that HIV protease inhibitors (saquinavir, ritonavir, indinavir, nelfinavir, amprenavir, darunavir, GS-8374, atazanavir, brecanavir, and acetyl-pepstatin) do not bind to the hDdi2 RVP domain (Figure S15). Thus, we hypothesized that hDdi2 is either catalytically inactive or requires some stimulus or protein partner for its activation.

## Discussion

We report here the first structural and functional analysis of mammalian Ddi1-like protein, human Ddi2. The Ddi1-like protein family is intricately connected to the UBQ-proteasome pathway, as its UBL domain interacts both with the proteasome and UBQ and its UBA interacts with UBQ and UBQ-chains^11^,^13^,^24^. Based on sequence analysis and genomic organization, we suggest, that hDdi2 is the original version of yDdi1 and non-mammalian orthologs of Ddi1-like proteins. Strikingly, hDdi2 differs from yDdi1 on several levels. One obvious difference is the loss of the UBA domain at the hDdi2 C-terminus. Therefore, we inspected hDdi2 for another potential UBQ-interacting motif (*-**L-**X-X-**A-**X-X-X-**S**-*), which we subsequently identified at the C-terminus (*-**L-**A-E-**A-**L-Q-K-**S**-*). We applied NMR chemical shift perturbation analysis to reveal that UBQ binds to hDdi2 C-terminal UIM specifically, but with a K_d_ of 2.2–3.2 mM. It will be interesting to explore whether such binding has any physiological relevance.

Recent work by Nowicka and co-workers showed that the yDdi1 UBL domain can bind UBQ^13^. This surprising feature completely changed our view of the Ddi1-like protein acting as a classical shuttle, suggesting that it may have an alternative mechanism. Therefore, we inspected hDdi2 UBL for its structural and functional properties. Our NMR structure of hDdi2 UBL indicates that unlike the positively charged β-sheet interaction area of yDdi1 UBL, which is complementary to the UBQ patch, the hDdi2 UBL has a small hydrophobic area that is moderately charged. Due to dissimilar charge distribution on the interaction patch, the pattern of interaction partners might differ. This assumption supports the NMR CSP analysis of hDdi2 UBL and UBQ, which shows weak but specific interaction between these two proteins (K_d_ of 0.42–1.1 mM).

Prompted by the above findings, we subjected hDdi2 to pull-down experiments with all eight native di-ubiquitin conjugates. We assumed that, if the observed weak hDdi2-UBQ affinity has any significance within the cell, an increase in affinity towards some of the UBQ chains would be observed. Notably, neither FLAG-tagged hDdi2 nor HA-tagged hDdi2 were able to pull down any di-UBQs. These results indicate significant differences between hDdi2 and yDdi1.

Yet another interesting feature of all Ddi1-like proteins is the presence of a highly conserved RVP domain, the function of which is largely unresolved. We solved the X-ray structure of hDdi2 RVP and compared it with yDdi1 RVP. As expected, both RVPs are structurally almost identical and quite similar to HIV-1 protease. The structural conservation of the catalytic residues indicates that it could be proteolytically active, although the catalytic cavity is significantly larger than those of other retroviral proteases and might accommodate even small proteins. While some work indicates that leishmanial Ddi1 is catalytically active at acidic pH and cleaves HIV substrates and BSA, we could not confirm these findings with hDdi2 using an HPLC-based method (see Figure S11). Moreover, we did not detect any putative proteolytic activity of hDdi2 with peptide-derived HIV-1 substrates and other proteins. In addition, PICS with an HEK293-derived peptide library revealed no cleavage connected to hDdi2. We also found that no HIV protease inhibitors bind to the RVP domain, as monitored by ITC. From these data, we infer that the RVP domain of hDdi2 likely does not possess intrinsic proteolytic activity. On the other hand, recent data suggests a potential hydrolytic function of RVP that is important for *Drosophila* development and is dependent on intact RVP^20^. That led us to hypothesize that the hDdi2 RVP domain may become catalytically active in more complex arrangement with yet to be identified protein partner.

The identification of the hDdi2 HDD domain goes in line with our hypothesis. This helical arrangement precedes RVP in most Ddi1-like orthologs, suggesting its functional importance. We determined the solution structure of hDdi2 HDD. It consists of a globular arrangement of 4 α-helices and shares broad similarities with the Sti1-like domain of Rad23, which is not structurally similar to any other known protein. All helices pack against each other and form a compact bundle with a hydrophobic core. This bundle superimposes well with the N-terminal part of yDdi1 HDD (identification and structurally characterization of which are described in our back-to-back publication^23^), which may suggest a similar function. Whether HDD could act as an interaction platform for an RVP substrate remains to be determined.

Overall, we present the first detailed study of hDdi2. We determined the 3D structures of all individual protein domains, including the previously unknown helical domain of hDdi2 (HDD). We also identified a novel UBQ-interacting motif (UIM) at the C-terminus of hDdi2. Furthermore, we show that the *in vitro* binding of mono-UBQ to its cognate domains is very weak but specific. We did not observe any binding of any native di-ubiquitin conjugates, which makes hDdi2 unique and diverse from yDdi1. Moreover, we thoroughly studied the RVP domain of hDdi2, solved its 3D structure by protein crystallography, and showed that it is homologous to yDdi1 RVP and HIV-1 protease. It remains to be determined whether RVP processes any substrates in a cellular context, perhaps after activation by a yet-to-be-identified stimulus or protein partner, or whether it exerts a different structural or functional role not directly linked to peptide bond hydrolysis.

## Methods

### Protein expression and purification

All proteins, including full-length hDdi2 and its truncated forms (UBL, residues 1–76; HDD, residues 116–212; RVP, residues 212–360; HDD-RVP, residues 116–360; RVP full-C, residues 212–399; and hDdi2 ΔUIM, residues 1–360), human ubiquitin, and Nedd8, were cloned into the vector pET16b (Novagen) in-frame with an N-terminal histidine tag (Fig. 1B). HDD was expressed in fusion with SUMO at the N-terminus. All constructs were expressed in *E. coli* BL21(DE3)RIL host cells; subsequently resuspended in buffer containing 50 mM Tris-HCl, pH 8.0, 50 mM NaCl, and 1 mM EDTA; and lysed by three passages through an EmulsiFlex-C3 high pressure homogenizer (Avestin, Canada) at 1200 bar. Proteins were purified using nickel affinity chromatography and eluted with 250 mM imidazole. Proteins were then dialyzed overnight into 50 mM HEPES, pH 7.4, 150 mM NaCl, and 10% glycerol and applied onto a Superdex 75 or 200 16/60 gel filtration column (GE Healthcare), depending on the protein mass. Individual fractions were analyzed by SDS-PAGE and/or Western blot.

For NMR experiments, hDdi2 UBL, hDdi2 HDD, hDdi2 RVP full-C, and human ubiquitin were expressed as ^15^N- and ^15^N/^13^C-labeled proteins; Nedd8 was expressed as an ^15^N-labeled protein. Cells were grown in minimal medium containing 0.8 g/l [^15^N]ammonium chloride and 2 g/l d-[^13^C]glucose, as required. Further procedures were the same as mentioned above, except the size-exclusion chromatography was carried out in buffers used for NMR titrations.

### Mammalian-expressed protein immobilization for PICS assay

For PICS proteolytic activity experiments and pull-downs, DNA encoding both N- and C-terminally FLAG-tagged full-length hDdi2 were cloned into the pTRE-Tight vector, and the constructs were transfected into HEK293A2 cells grown on DMEM media, using lipofectamine to produce a stable transfected cell line. Clones with a high level of FLAG-hDdi2 expression were selected by Western blot. Cells from ten 100-mm cell culture dishes were harvested by washing into PBS followed by centrifugation (2 min, 225 g, RT) and washed 3x with PBS. Cells were resuspended in ice cold lysis buffer (50 mM HEPES. pH 7.8, 150 mM NaCl, 0.4% Igepal CA-630) and lysed on ice using 3 freeze/thaw cycles on dry ice, each followed by repeated aspiration of the cell suspension with a 30-gauge needle. The cell lysate was diluted 4x with lysis buffer without Igepal and cleared by centrifugation (15 min, 20,000 g, 4 °C). Supernatant was loaded on M2 anti-FLAG magnetic beads (Sigma-Aldrich) in batch format according to the manufacturer’s recommendation. After a 1-h equilibration, beads were washed 4 times with PBS. The purification process and the final amount and purity of protein immobilized on magnetic beads were monitored by SDS-PAGE and Western blot. FLAG-tagged hDdi2 immobilized on magnetic beads was subsequently used for PICS experiments. As control samples, an identical amount of magnetic beads was incubated with an equal (in protein mass) amount of cell lysate from non-transfected cells and processed the same way.

### Pull-down experiments

Beads with approximately 3 μg of hDdi2 immobilized *via* FLAG-tag on either the N- or C-terminus were equilibrated with UBQ-binding buffer (PBS, pH 7.4, 1% Triton X-100, 5 mM EDTA, 0.2 mg/ml BSA) and mixed with 1 μg of di-ubiquitin conjugate (UbiQ) of given linkage type in a total volume of 50 μl of the same buffer. The final mixture was incubated for 2 h at room temperature with mild agitation. Beads were washed twice with 150 μl and 100 μl of TBS, and bound proteins were eluted by heating to 95 °C for 3 min in 5 μl of 2x non-reducing SDS-PAGE sample buffer (125 mM Tris, pH 6.8, 4% SDS, 20% (v/v) glycerol, 0.004% bromphenol blue). The whole eluted fraction was separated by 18% Tris-glycine SDS-PAGE and blotted onto a PVDF membrane. The membrane was denatured (6 M guanidium chloride, 20 mM Tris, pH 7.5, 1 mM PMSF, 5 mM β-mercaptoethanol) and developed using anti-ubiquitin rabbit polyclonal antibody (Dako). Experiments were performed with di-ubiquitins of all eight native linkage types. In addition, potential binding was tested also with Lys48- and Lys63-linked chains synthetized in house according to Pickart and co-workers^41^. Negative controls with either no immobilized hDdi2 protein or without loaded di-ubiquitin were treated the same way.

### X-ray crystallography

Crystals of hDdi2 RVP were grown by the hanging drop vapor diffusion technique at 19 °C with 0.2 M ammonium acetate, 0.1 M Bis-Tris, pH 5.5, and 25% PEG 3350 as precipitant. For cryoprotection, crystals were soaked in the reservoir solution supplemented with 25% (v/v) glycerol. Diffraction data were collected at 100 K at BESSY beamline 14.2 at the Hemholtz Zentrum Berlin, Germany^42^. Data were integrated using Mosflm v7.0.6 and later scaled with SCALA v3.3.20^43^,^44^. The crystal structure was solved by molecular replacement using the program Molrep and the structure of yDdi1 RVP (PDB code 2I1A) as a template^22^,^45^. Model refinement was carried out with REFMAC 5.6 from the CCP4 package^46^,^47^, interspersed with manual adjustments using Coot^48^. Atomic coordinates and experimental structure factors have been deposited in the Protein Data Bank under the code 4RGH. Data collection and refinement statistics are given in Table S2.

### Peptide synthesis

The UIM peptides (hDdi2 C-terminus-derived UIM of amino acid sequence EEIADQELAEALQKSAEDAE and its scrambled version AELEQIAEDALEKEDSQEAA) were synthetized on an ABI 433A solid phase synthesizer (Applied Biosystems, USA) at the peptide synthesis core facility of IOCB, Czech Republic. They were further purified in the form of C-terminal amides by reverse-phase high-performance liquid chromatography (HPLC) on a semipreparative C18 column (Labio a.s., Prague, Czech Republic). Purified fractions were frozen in liquid nitrogen, lyophilized, and dissolved in DMSO prior to further use.

### Nuclear magnetic resonance spectroscopy

NMR spectra for interaction site identification were acquired from 350 μl samples of 0.1 mM (peptide binding) or 0.05 mM (protein – protein interaction) ^15^N-labeled hDdi2 UBL and hDdi2 RVP full-C in 50 mM sodium phosphate buffer, pH 7.4, and from 0.1 or 0.05 mM UBQ in 50 mM sodium phosphate buffer, pH 6.0 and pH 7.4. All buffers contained 5% D_2_O/95% H_2_O. Spectra for structural determination and backbone assignments were acquired at 0.5 mM concentration of ^13^C/^15^N-labeled proteins. NMR data were collected at 25 °C on 600 and 850 MHz Bruker Avance spectrometers equipped with triple resonance (^15^N/^13^C/^1^H) cryoprobes. Resonance assignments were obtained using a previously published approach^49^,^50^. Detailed experimental procedures for all the NMR measurements, structure calculations, and chemical shift mapping are described in the Supplementary Information.

### Small-angle X-ray scattering

The His-tagged HDD-RVP construct of hDdi2 (residues 116–360) was purified and concentrated in SAXS buffer (25 mM Tris/HCl, 75 mM NaCl, 5% glycerol, 1 mM DTT, pH 7.4). A series of dilutions (10, 5, and 2.5 mg/ml) and buffer alone were frozen and shipped to the SIBYLS facility at the Advanced Light Source (ALS) for automated SAXS analysis as described^51^. SAXS data were acquired for 0.5, 1, 2, and 4 sec for each sample. Due to a slight concentration-dependent effect in the low-q region, the data at 10 mg/ml were discarded. The 5 and 2.5 mg/ml data were merged for data analysis using the ATSAS software suite^52^. The molecular weight was calculated using the *Qr* method as described^53^. BUNCH software was used for modeling, using the crystal structure of the RVP domain (residues 231–360) with a fixed P2 symmetry axis and the NMR structure of the HDD domain (residues 131–190). Twenty models were calculated with χ^2^ fit to experimental data ranging between 1.64 and 2.39, and averaged using DAMAVER. The resulting bead model was converted into a volumetric map using the program SITUS and visualized in Chimera^54^,^55^.

### Isothermal titration calorimetry (ITC)

The ability of hDdi2 RVP to bind HIV-1 protease inhibitors was analyzed at 25 °C using a high-throughput screening Auto-iTC_200_ system (MicroCal, GE Healthcare Life Sciences). Aliquots (2 μl) of 120 μM protease inhibitors (saquinavir, ritonavir, indinavir, nelfinavir, amprenavir, darunavir, GS-8374, atazanavir, brecanavir, and acetyl-pepstatin) were injected stepwise into a sample cell containing 200 μl of 10 μM hDdi2 RVP (concentration calculated based on the molecular weight of the dimer; HPLC amino acid analysis was performed). The titrations were monitored by MicroCal software implemented in Origin 7.0 (MicroCal, GE Healthcare Life Sciences).

### PICS assay

A HEK293-cell-derived peptide library for PICS experiments was prepared as described by Schilling *et al.*^56^. Isolated denatured proteins were cleaved into peptides using trypsin (Sigma Aldrich) and GluC as working proteases. After abolishing the working protease activity using PMSF, a second round of sulfhydryl reduction and alkylation was performed, and primary amines on peptide N-termini and lysine side chains were blocked using formaldehyde-cyanoborohydride reductive dimethylation. Excess modification reagents were removed by gel filtration, and the peptide library was purified and transferred to HPLC grade water using a Sep-Pak Plus C-18 solid phase extraction cartridge (Waters), following the manufacturer’s protocol. The peptide concentration in the library was adjusted to 2 mg/ml. The integrity of the peptide library was confirmed by LC-MS/MS analysis. The final amine-protected mammalian proteome-derived peptide library was stored in aliquots at −80 °C until further use.

For the endopeptidase assay, peptide library (1 mg/ml) was incubated in 200 μl of 100 mM sodium acetate, 300 mM NaCl, pH 4.0, with 4 μg of recombinant full-length hDdi2. Reactions were incubated for 12 h at 37 °C, then heat-inactivated for 30 min at 70 °C and transferred to 200 mM HEPES, pH 8.0, using a Sep-Pak Light C-18 solid phase extraction cartridge (Waters), following the manufacturer’s protocol.

Subsequently, newly formed peptide free N-termini (products of proteolytic cleavage) were biotinylated *in vitro* by incubation with 350 μM sulfo-NHS-SS-biotin (Thermo-Scientific) for 4 h at room temperature. Biotinylated products were then immobilized on streptavidin agarose (Solulink) by 2 h incubation with mild agitation at room temperature, followed by washing. Additional washing steps (2 M urea followed by 20% isopropanol, 5% DMSO, and finally 5% acetonitrile, all in washing buffer [50 mM HEPES, pH 7.5, 150 mM NaCl]) were added into the protocol, followed by ten washes with washing buffer alone. Immobilized peptides were eluted with 20 mM DTT and desalted using Pepclean C-18 reverse phase cartridges (Thermo Scientific), following the manufacturer’s protocol, and analyzed by mass spectrometry.

As negative controls, we used D252A hDdi2 and a mock reaction with buffer added instead of enzyme. As a positive control, the HIV-1 protease cleavage profile in 100 mM Na acetate, 300 mM NaCl, pH 4.7, was tested using wild-type HIV-1 protease and a catalytically inactive mutant (D25N) in a 1:200 protease-to-library ratio. The proteolytic cleavage assay was carried out in 100 mM sodium acetate, 300 mM NaCl, pH 5.0, and 100 mM HEPES, 300 mM NaCl, pH 7.0, with processing and control reactions as described above.

Eukaryotic-expressed hDdi2 was also tested in the PICS assay. For those experiments, magnetic beads with immobilized FLAG-tagged hDdi2 in an amount corresponding to approximately 1 μg of immobilized protein (based on Western blot) were mixed with 200 μg of the peptide library. After a 12-h incubation at 37 °C, beads were magnetically removed, residual protein was heat-inactivated, and the sample was further processed as described above. This assay was carried out under three different buffer conditions (100 mM sodium acetate, 300 mM NaCl, pH 4.0; 100 mM sodium acetate, 300 mM NaCl, pH 5.0; and 100 mM HEPES, 300 mM NaCl, pH 7.0). As a control, an identical amount of magnetic beads incubated with the lysate of nontransfected cells was used.

### Data analysis of the PICS assay

Data were analyzed by a series of predesigned queries in Microsoft Access database management software. First, lists of identified peptides from each MS run were loaded to the database and filtered for peptides containing products of N-terminal modification by biotinylation. Second, peptides with over 80% confidence were picked for the enzyme tested (hDdi2 or HIV-1 protease), while peptides with over 10% confidence were picked for control reactions (catalytically inactive mutants and mock reactions). To properly subtract the background signal, the list of peptides identified in the tested enzyme reaction was screened for peptides presented in the mock reaction as well as in the reaction with catalytically inactive enzyme (hDdi2 [D252A] or HIV-1 protease [D25N]), and those peptides were removed from processing. Finally, the tested enzyme reactions were screened for peptides identified in the original unprocessed peptide library. Such peptides were then removed from the analysis.

The final cleared list of identified peptides was then mapped on the FASTA database used for proteomics database searching. By alignment of identified peptides with the database, the N-terminal portions of cleaved peptides (preceding the cleavage site) were determined. If multiple computationally identified preceding sequences were found for one identified peptide, they were removed from processing, while the identified peptide sequences were kept in the list for downstream analysis. The final list of substrate peptides containing sequences of four P-prime amino acids identified by MS and four P amino acids identified computationally was then created. The frequency of each amino acid in each particular position was calculated and plotted, yielding the substrate specificity matrix for the enzyme studied.

### HPLC analysis

The hydrolysis of peptides corresponding to the HIV-1 Gag and Gag-Pol processing sites was performed in 50 mM sodium acetate, pH 5.0, containing 0.5 M NaCl and 2 mM EDTA, using 200 μM peptide and 75 nM hDdi2 RVP expressed in a prokaryotic system. Additionally, cleavage of 5 μM proteins (bovine serum albumin, human serum albumin, bovine casein, and bovine insulin) in 100 mM sodium acetate, pH 5.0, 1 M NaCl, and 4 mM EDTA using 200 nM hDdi2 RVP was monitored. The reaction mixture was incubated at 37 °C for 24 h. The reactions were stopped by addition of formic acid to a final concentration of 20% (v/v). Aliquots (5 μl) of the reaction mixtures were subsequently injected into a Zorbac SB-C18 reversed-phase chromatography column (Agilent), and peptides were resolved using a water-acetonitrile gradient on a high-performance liquid chromatograph (HPLC) (Agilent). The peptide cleavage was monitored at 220 nm.

## Additional Information

**Accession codes**: Coordinates and structure factors for the RVP crystal structure were deposited in the PDB under accession code 4RGH. The structure and assigned chemical shifts for the UBL domain of hDdi2 were deposited in the PDB and BMRB databases under accession codes 2N7D and 25801, respectively. The structure and assigned chemicals shifts for the HDD of hDdi2 were deposited in the PDB and BMRB databases under accession codes 5K57 and 30097, respectively.

**How to cite this article**: Sivá, M. *et al.* Human DNA-Damage-Inducible 2 Protein Is Structurally and Functionally Distinct from Its Yeast Ortholog. *Sci. Rep.* **6**, 30443; doi: 10.1038/srep30443 (2016).

## Supplementary Material

Supplementary Information
