# Supplementary material for: Human DNA-Damage-Inducible 2 Protein Is Structurally and Functionally Distinct from Its Yeast Ortholog
Source: Sci Rep. 2016 Jul 27;6:30443. doi: 10.1038/srep30443 (PMC4962041; doi:10.1038/srep30443)
Supplement: Supplementary Information [file srep30443-s1.pdf]

Human DNA-Damage-Inducible 2 Protein Is Structurally and Functionally Distinct from Its Yeast Ortholog

Monika Sivá[1,2#], Michal Svoboda[1,3#], Václav Veverka[1], Jean-François Trempe[4], Kay Hofmann[5], Milan Kožíšek[1], Rozálie Hexnerová[1], František Sedlák[1,2], Jan Belza[1,6], Jiří Brynda[1], Pavel Šácha[1], Martin Hubálek[1], Jana Starková[1], Iva Flaisigová[1], Jan Konvalinka[1,6] and Klára Grantz Šašková[1,6*]

[1]Gilead Sciences and IOCB Research Center, Institute of Organic Chemistry and Biochemistry of the Academy of Sciences of the Czech Republic, Flemingovo n. 2, 166 10 Prague 6, Czech Republic

[2]First Faculty of Medicine, Charles University in Prague, Katerinska 32, 121 08, Prague 2, Czech Republic

[3]Department of Physical and Macromolecular Chemistry, Faculty of Science, Charles University, Hlavova 8, 120 00 Prague 2, Czech Republic

[4]Groupe de Recherche Axé sur la Structure des Protéines, Department of Pharmacology & Therapeutics McGill University, Montreal, QC, H3G 1Y6, Canada

[5]Institute for Genetics, University of Cologne, Zülpicher Str. 47a, 50647 Cologne, Germany

[6]Department of Biochemistry, Faculty of Science, Charles University, Hlavova 8, 120 00 Prague 2, Czech Republic

*Correspondence: saskova@uochb.cas.cz

# Authors contributed equally to this work.

Results

NMR interaction studies of hDdi2

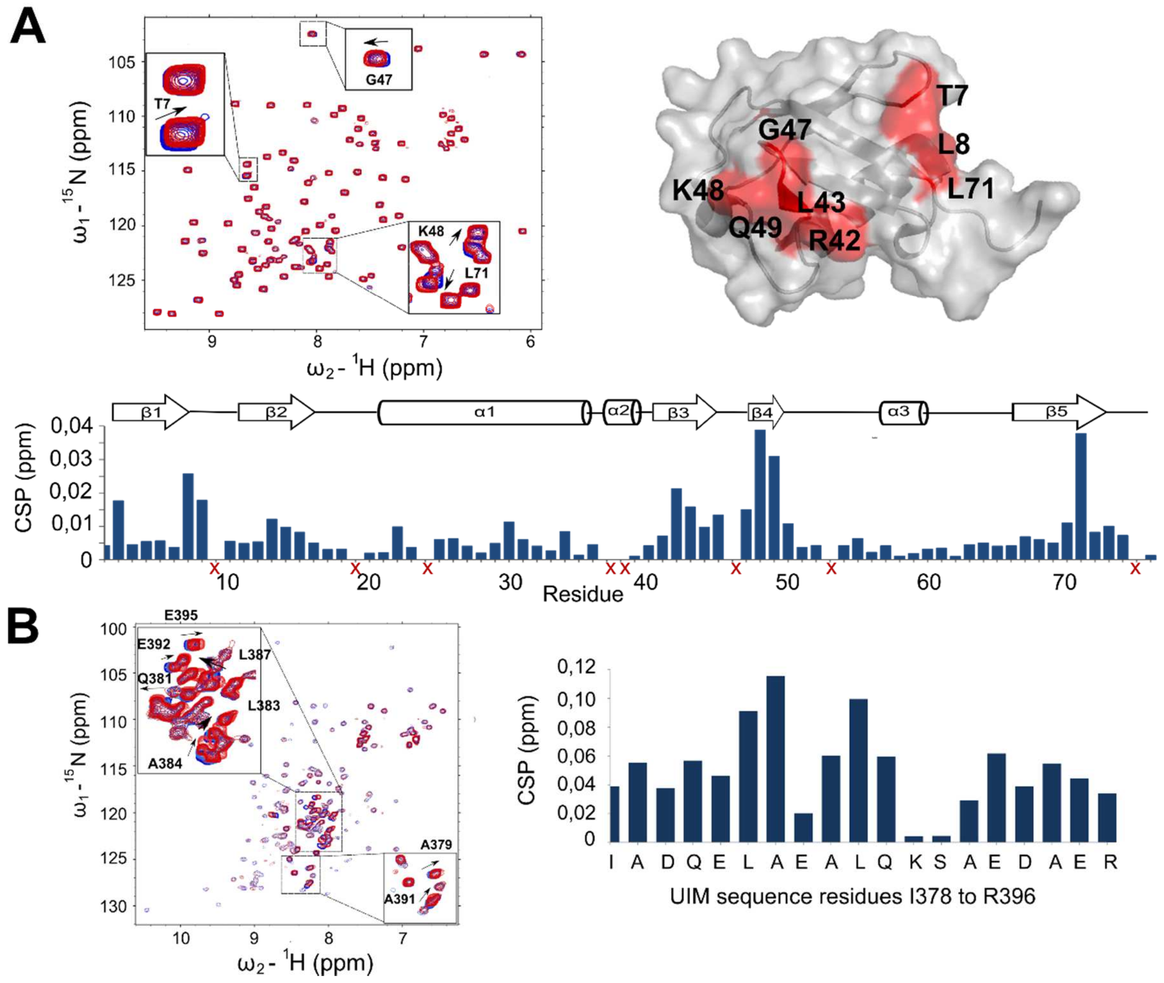

Figure S1: UBQ - hDdi2 UIM interaction verification. A) Upper left: HSQC spectra of ^{15}N-labeled UBQ (blue) with 5-fold molar addition of non-labeled hDdi2 RVP full-C (red) reveals specific but very weak CSPs of UBQ backbone amide signals (below). The shifts are mapped onto the UBQ structure (PDB entry 1D3Z)[3] (upper right). **B)** Reverse mapping of the interaction of Ddi2 UIM with UBQ on HSQC spectra of ^{15}N-labeled hDdi2 RVP full C (blue) with 5-fold molar addition of non-labeled UBQ (red) (left). The plot of CSPs of the hDdi2-UIM sequence locus are shown on the right.

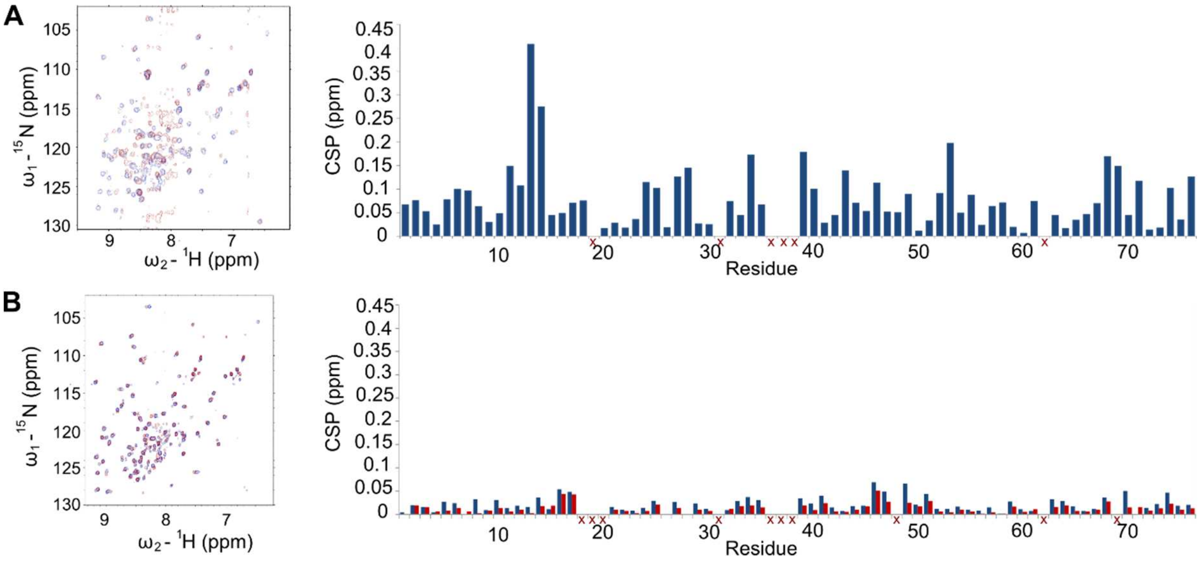

Figure S2: Interaction of Nedd8 with the C-terminal UIM motif of human Ddi2. The interaction of Nedd8 with UIM peptide was verified by acquisition of 2D HSQC spectra under two different conditions: **A)** 50 mM sodium acetate, pH 5, and **B)** 50 mM sodium phosphate, pH 7. Left: Alignments of spectra of ^{15}N-labeled Nedd8 without (blue) and with final addition of the ligand (red). Right: Plots of chemical shift perturbations upon peptide addition **A)** show remarkable shifts in several amino acids at pH 5 but **B)** no perturbation relevant for the interaction at pH 7, which is closer to physiological conditions. Moreover, addition of the scrambled peptide (red) did not change the profile of CSP on Nedd8 compared to the hDdi2 UIM peptide (blue). The interaction at pH 5 is electrostatic, and we propose that there is no physiological interaction between hDdi2 and Nedd8.

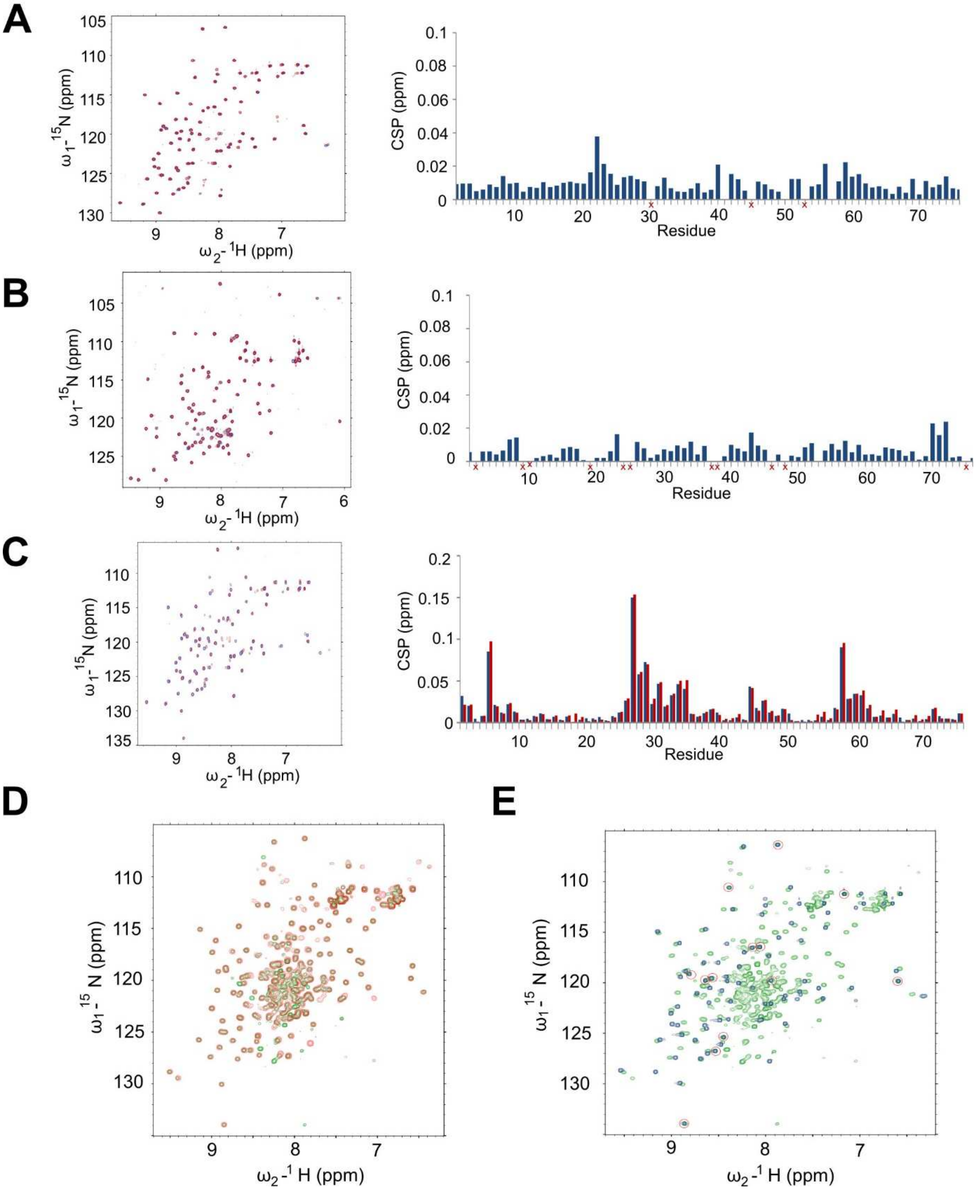

Figure S3: Human Ddi2 UBL domain interactions. A) hDdi2 UBL domain does not bind Nedd8. Left: 2D HSQC spectra of ^{15}N-labeled hDdi2 UBL domain (blue) upon 5-fold molar addition of Nedd8 (red). Right: Plot of CSPs of backbone amides observed after Nedd8 addition. **B)** UBQ does not bind to hDdi2 lacking N- and C-terminal domains. Left: No significant chemical shift perturbations were observed in 2D HSQC spectra of ^{15}N-labeled UBQ

(blue) upon 6-fold molar addition of hDdi2 HDD-RVP (red) lacking the N-terminal UBL domain and C-terminal UIM motif. Spectra monitoring changes in HSQC spectra of UBQ due to addition of hDdi2 with one of the terminal domains showed more significant CSP, assuming these domains bind UBQ *in vitro*, although very weakly. Right: Plot of CSPs of backbone amides observed in this experiment. **C)** The N-terminal UBL domain of hDdi2 does not bind C-terminal UIM peptide derived from hDdi2. Left: 2D HSQC spectra of 0.05 mM hDdi2 UBL before (blue) and after addition of the UIM peptide derived from the hDdi2 C-terminus (red) with final 1.9 mM concentration. Right: Plot of CSPs after addition of UIM-derived peptide (blue) and after addition of a scrambled peptide (red) to a final concentration of 1.2 mM. The interaction of the UBL domain with the peptides shows that the CSPs observed upon addition of UIM peptide are not sequence-specific but purely electrostatic. **D)** hDdi2 UBL domain does not bind the C-terminal UIM motif. 2D HSQC spectra of full-length hDdi2 protein (green) superimposed with 2D HSQC spectra of hDdi2 ΔUIM (red) exhibits no shifts in the signals of UBL domain, thus confirming the previous peptide titration experiments. **E)** hDdi2 UBL does not extend away from the body of the hDdi2 protein. 2D HSQC spectra of hDdi2 UBL (blue) superimposed with the HSQC of the full-length protein (green) show differences in signals of the UBL, suggesting a rather compact structure for the full-length protein. The few amino acids lacking the difference are marked with red circles.

Polyubiquitin chain binding is not preserved in human Ddi2

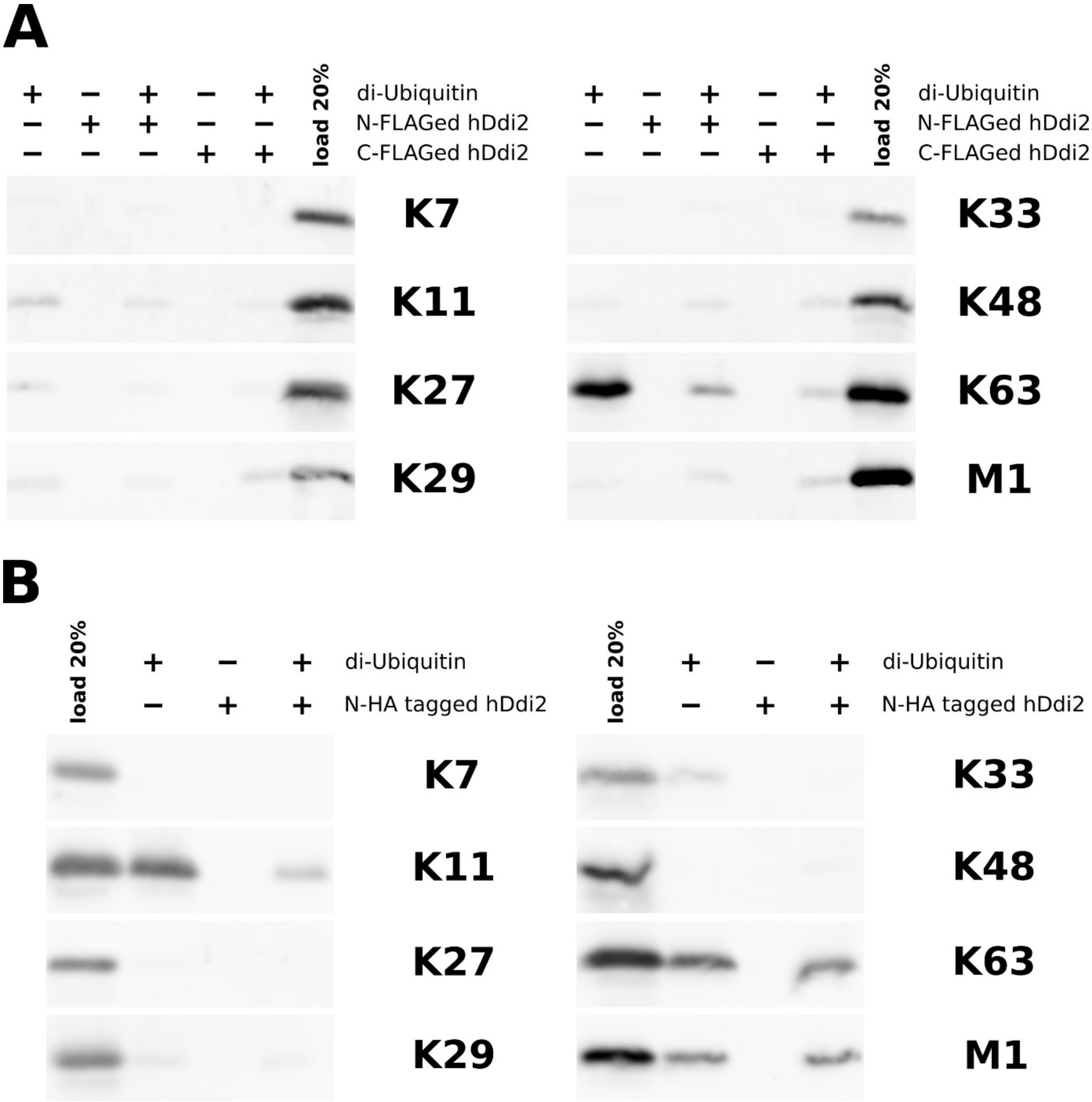

Figure S4: Polyubiquitin chain binding is not preserved in human Ddi2. Western blot analysis of pull-down experiments with di-ubiquitin conjugates of various linkage architecture. Human Ddi2 with a FLAG tag on either the N- or C-terminus **(A)** or an HA tag on the N-terminus **(B)** was immobilized on magnetic agarose beads. Beads were incubated with the di-ubiquitin conjugate of given linkage architecture (purchased from UbiQ, The Netherlands), washed, and eluted by boiling in non-reducing SDS sample buffer. Samples were analyzed on 18% SDS-PAGE followed by immunoblotting with anti-UBQ antibody (Dako).

Sti1-like domain identification

```
HsDdi2  ITSSPQGLDNPALLRDMLLAN....PHELSLLKERNPPLAEALLSGDLEKFSRVLVEQ 125-178
HsDdi1  KVAGLQGLGSPALIRSMLLSN....PHDLSLLKERNPPLAEALLSGSLETFSQVLMEQ
DmRingo DEFNVNFDDDPETVRQMFLSS....PETLSLLRQYNPSLAEAIDSGDKEKFARLLREH
SpMud1       MNNLTPENIRQTILAT....PFLLNRIRTEFPQLAAVL..NDPNAFATTWQSI
ScDdi1  DAATLSDEAFIEQFRQELLNN....QMLRSQLILQIPGLNDLV..NDPLLFRERLGPL
RAD23A  PLEFLRDQPQFQNMRQVIQQNPALLPALLQQLGQENPQLLQQI.SRHQEQFIQMLNEP
1oqy    HHHHHHHH HHHHHHHH        HHHHHHHH    HHHHHHHH   HHHHHHHHH
```

Figure S5: Sequence alignment of the human Ddi1-like proteins, *Drosophila melanogaster* Ddi1 (Rngo), *Saccharomyces pombe* Ddi1 (Mud1), *Saccharomyces cerevisiae* Ddi1, and RAD23A. For secondary structure prediction (H-helix, in red), the most closely related sequence from a known structure of human Rad23A was used (PDB 1oqy)[1].

Search for putative proteolytic activity and small-molecular binder of the RVP domain

PICS analysis:

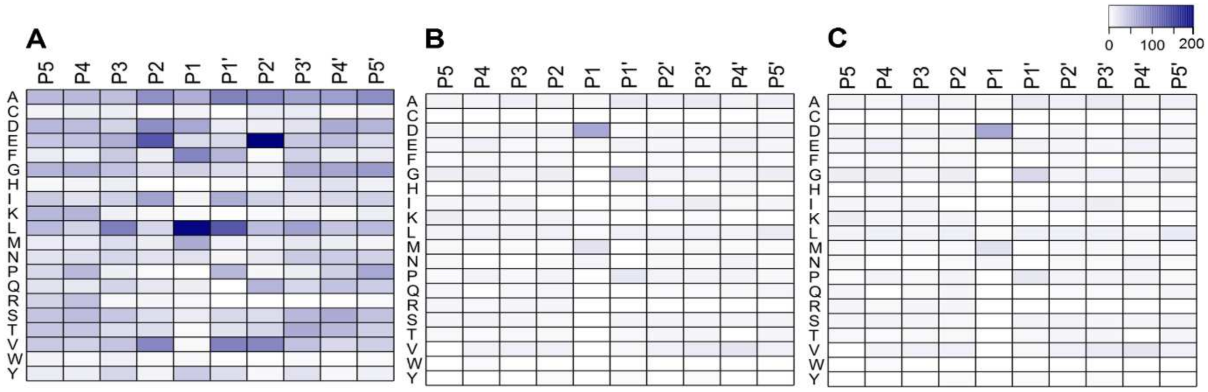

Figure S6: PICS (Proteomic Identification of Protease Substrates) results: Heatmap representation of protease substrate profile demonstrating the total counts of given amino acids (described by single letter code) per particular position (P5−P5´) in peptidic substrates in a HEK293 cell line-derived peptide library. The cleaved peptide bond is positioned between P1 and P1´. **A)** Library cleaved by HIV-1 protease (positive control) **B)** Library cleaved by hDdi2 protein in HEPES pH 7 (proteolytic activity assay). C) Background profile for the uncleaved library (negative control).

HPLC assay for human Ddi2 proteolytic activity

We used an HPLC assay to test putative hydrolysis of a complete set of HIV polyprotein-derived peptide substrates by hDdi2 RVP domain at pH 5.0 (and pH 7.0 for HIV substrates) and various salt concentrations (150 mM - 500 mM NaCl). We also used HPLC to assess putative cleavage of BSA, HSA, β-casein, and insulin by hDdi2 RVP domain.

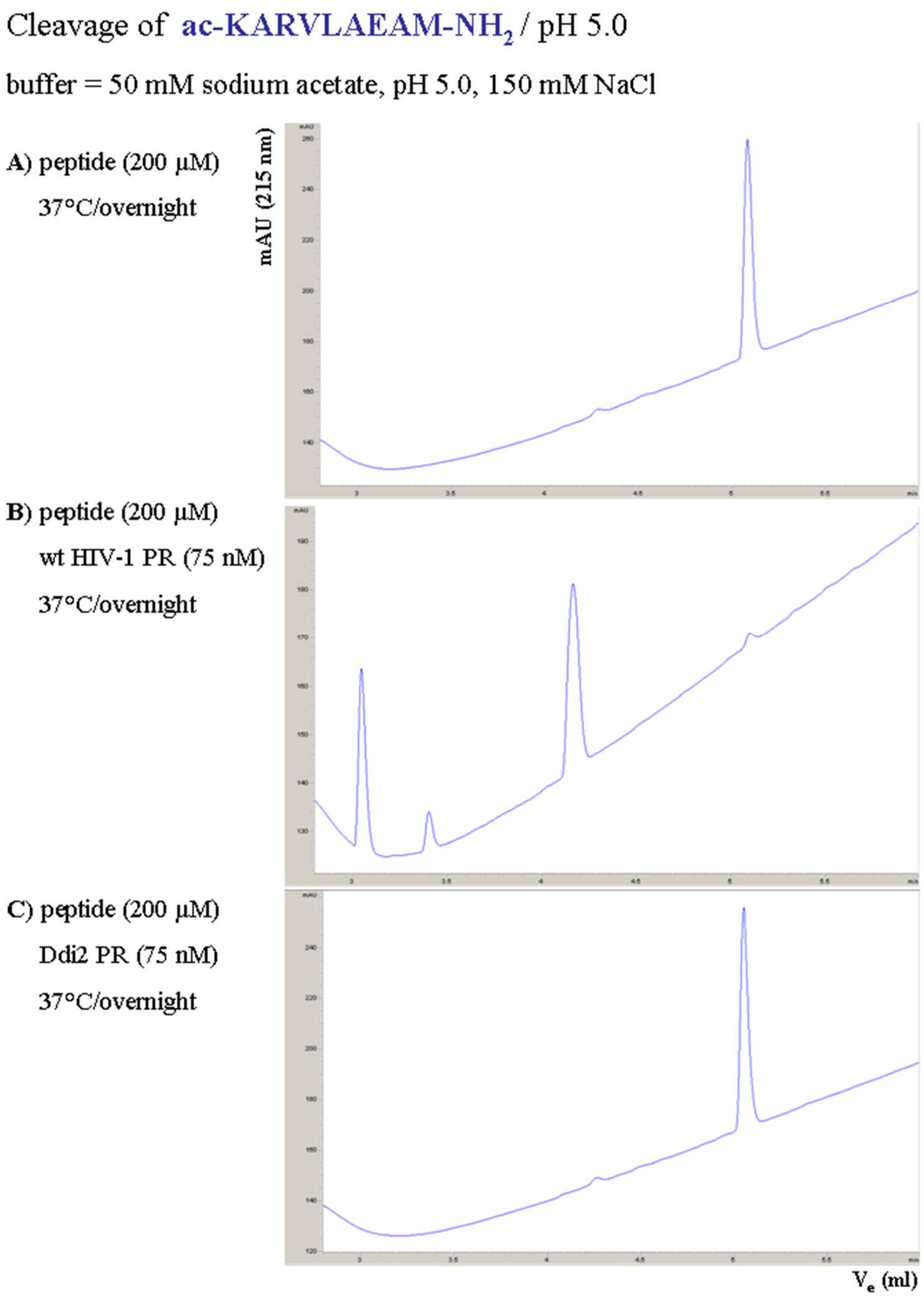

Figure S7: Testing of putative cleavage of an HIV polyprotein-derived peptide substrate with the amino acid sequence KARVLAEAM by hDdi2 RVP domain at pH 5.0 with 150 mM NaCl.

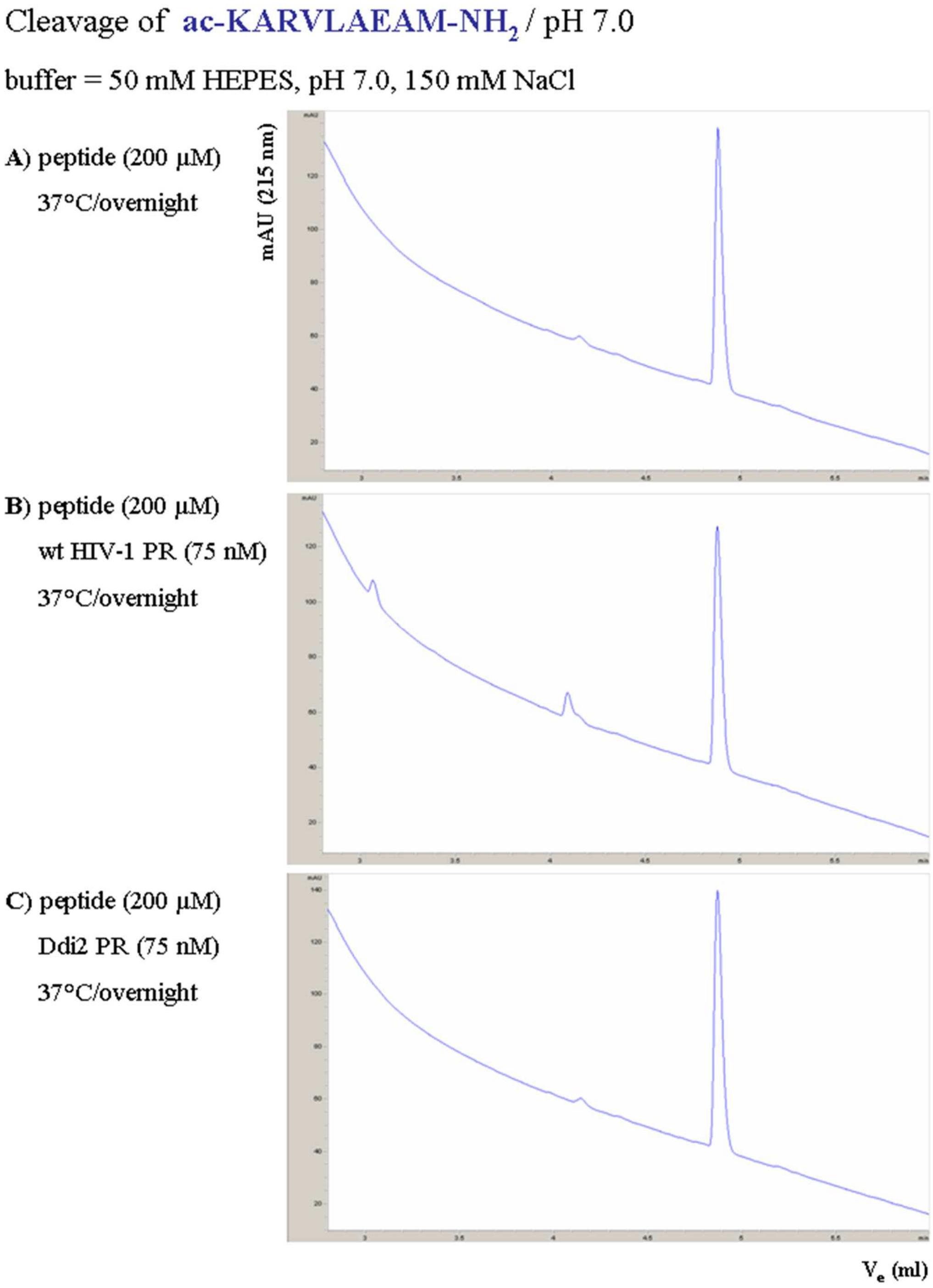

Figure S8: Testing of putative cleavage of an HIV polyprotein-derived peptide substrate with the amino acid sequence KARVLAEAM by hDdi2 RVP domain at pH 7.0 with 150 mM NaCl.

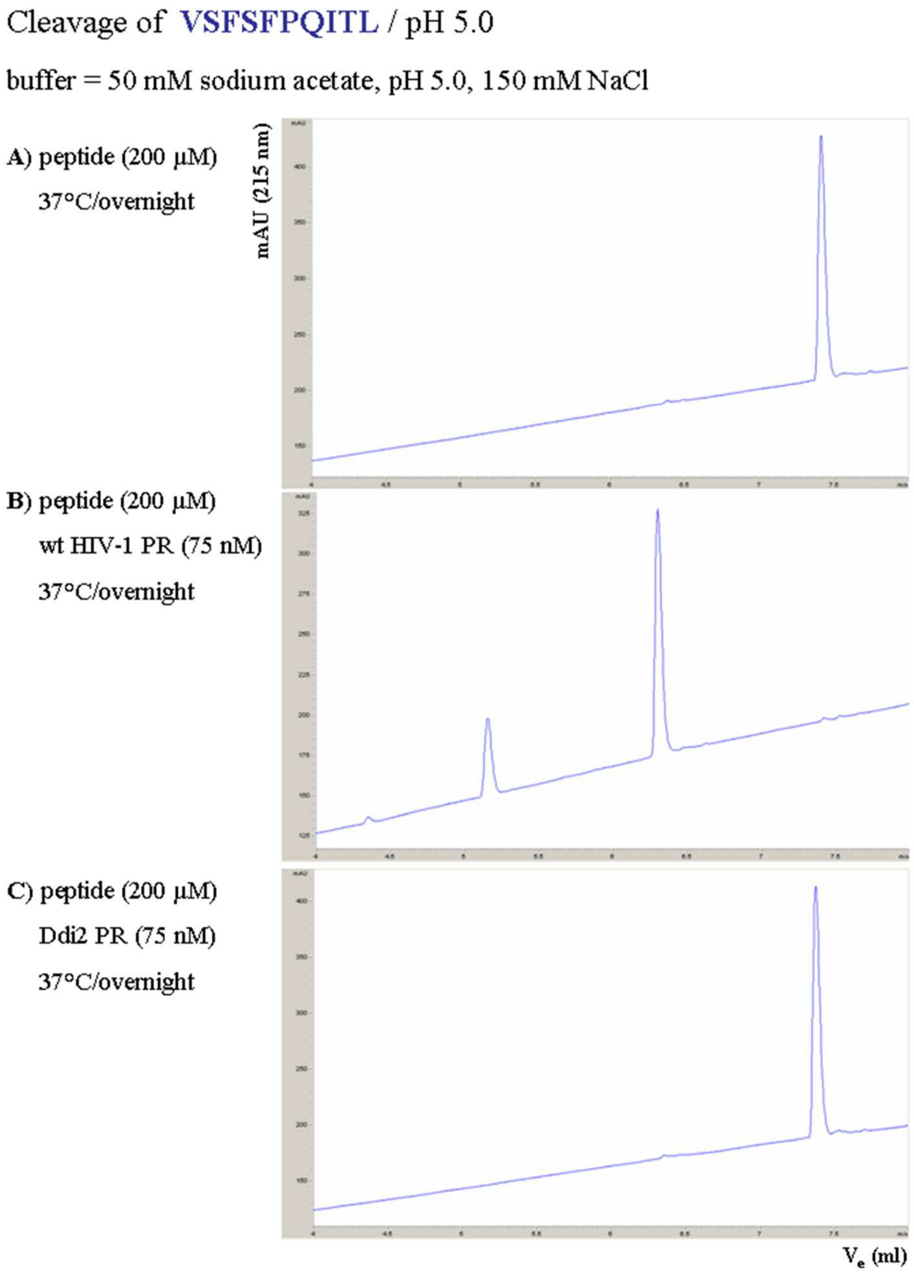

Figure S9: Testing of putative cleavage of an HIV polyprotein-derived peptide substrate with the amino acid sequence VSFSFPQITL by hDdi2 RVP domain at pH 5.0 with 150 mM NaCl.

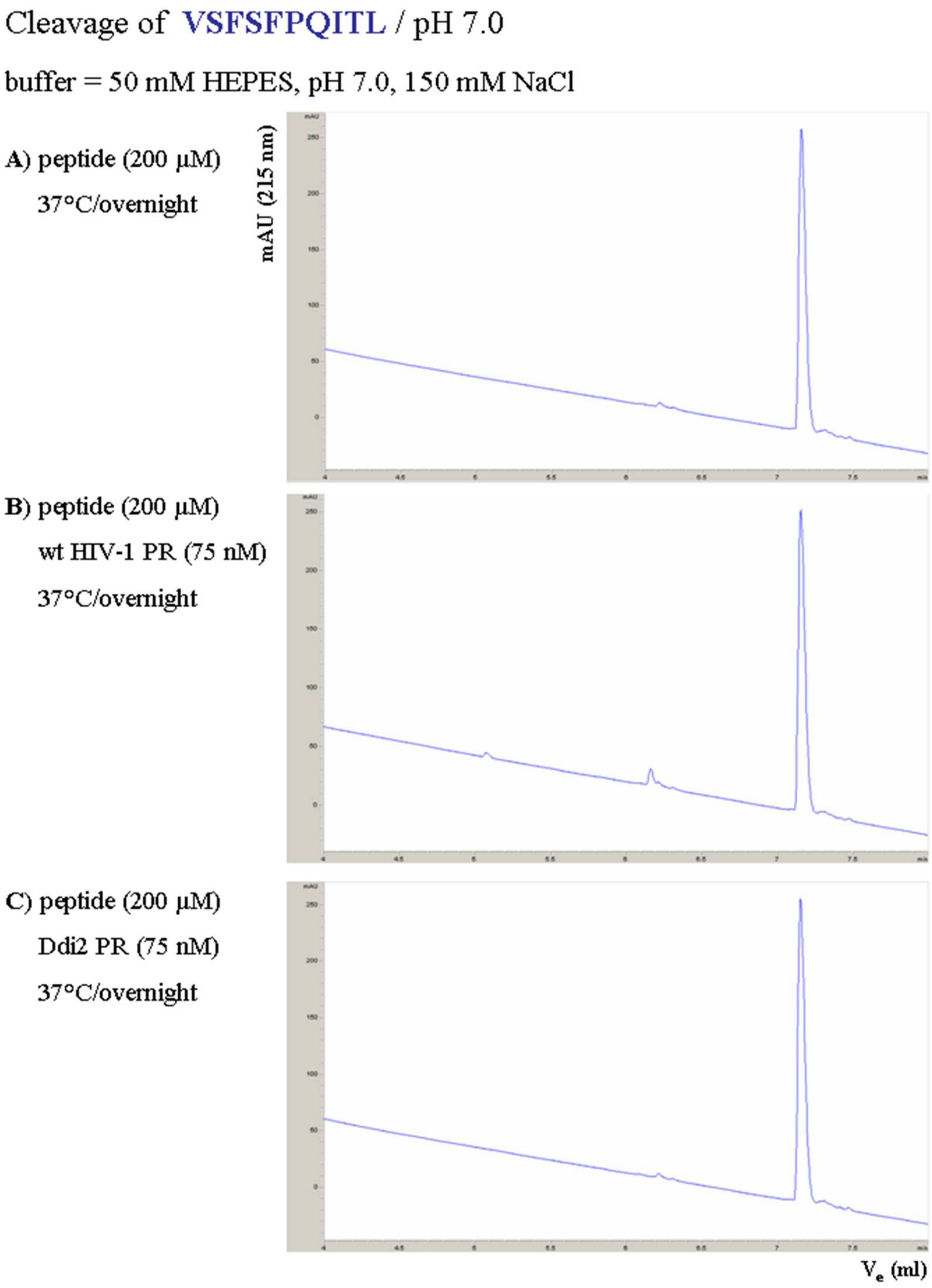

Figure S10: Testing of putative cleavage of an HIV polyprotein-derived peptide substrate with the amino acid sequence VSFSFPQITL by hDdi2 RVP domain at pH 7.0 with 150 mM NaCl.

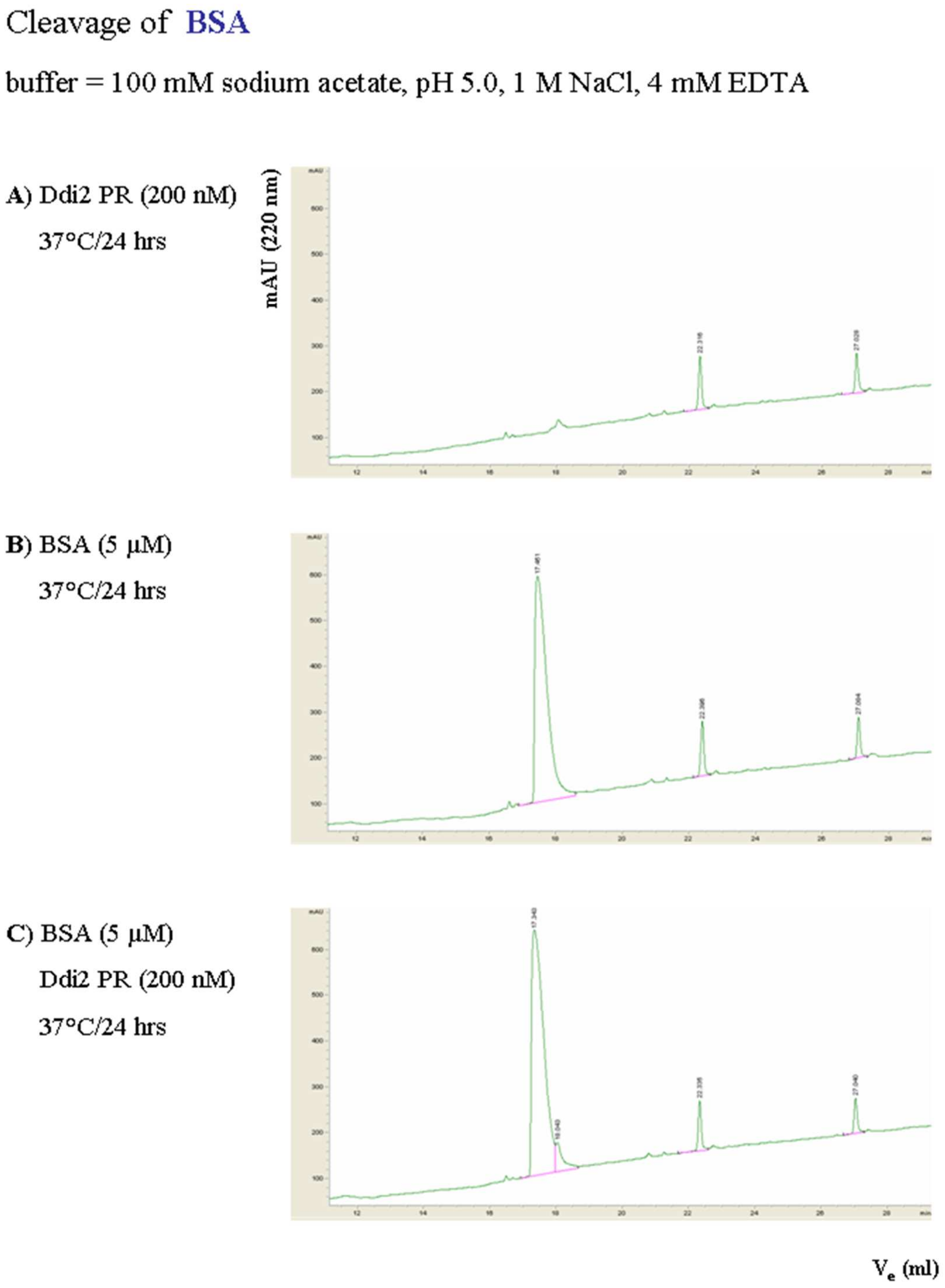

Figure S11: Testing of putative cleavage of bovine serum albumin (BSA) by hDdi2 RVP domain at pH 5.0 with 1 M NaCl.

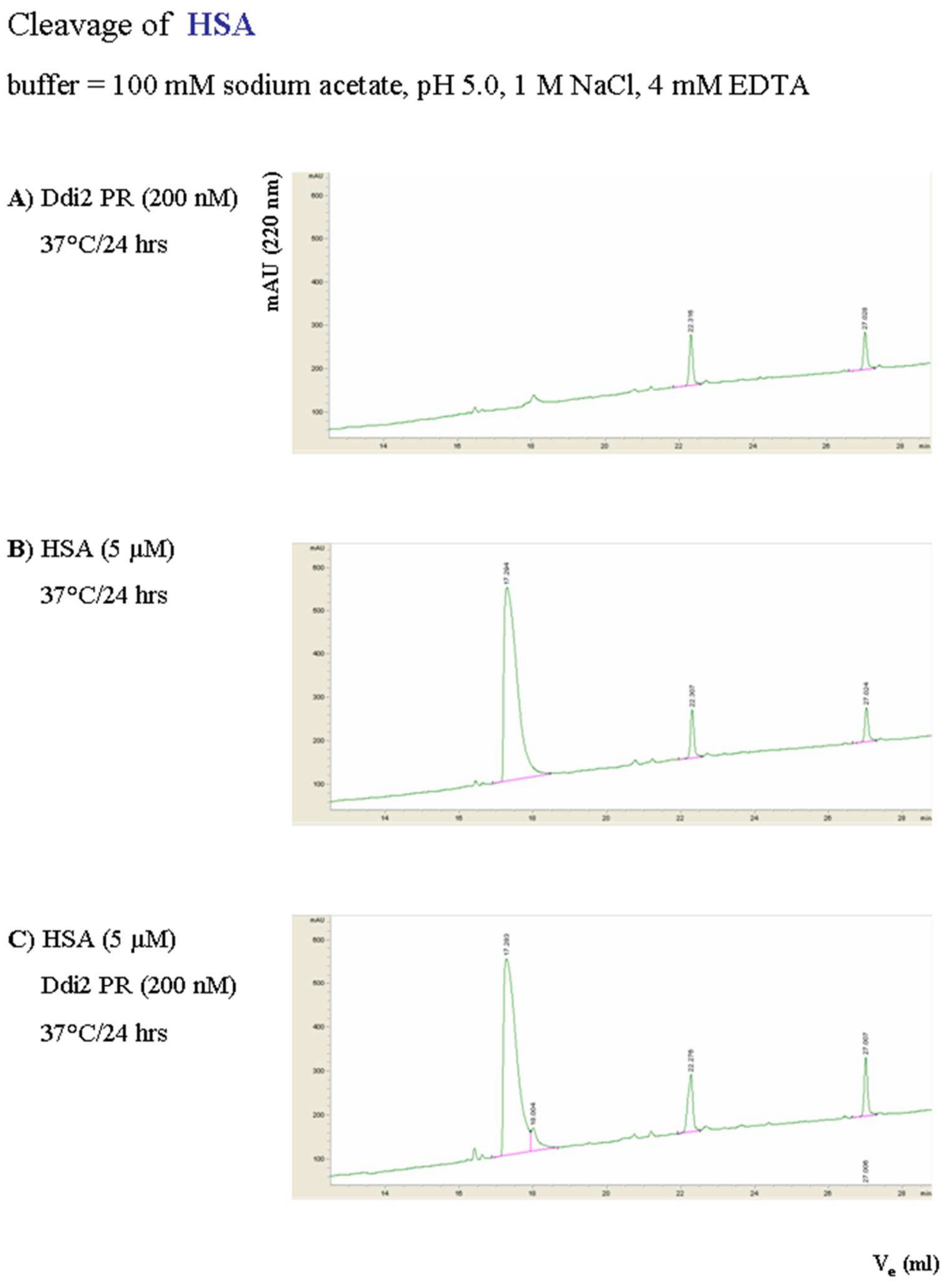

Figure S12: Testing of putative cleavage of human serum albumin (HSA) by hDdi2 RVP domain at pH 5.0 with 1 M NaCl.

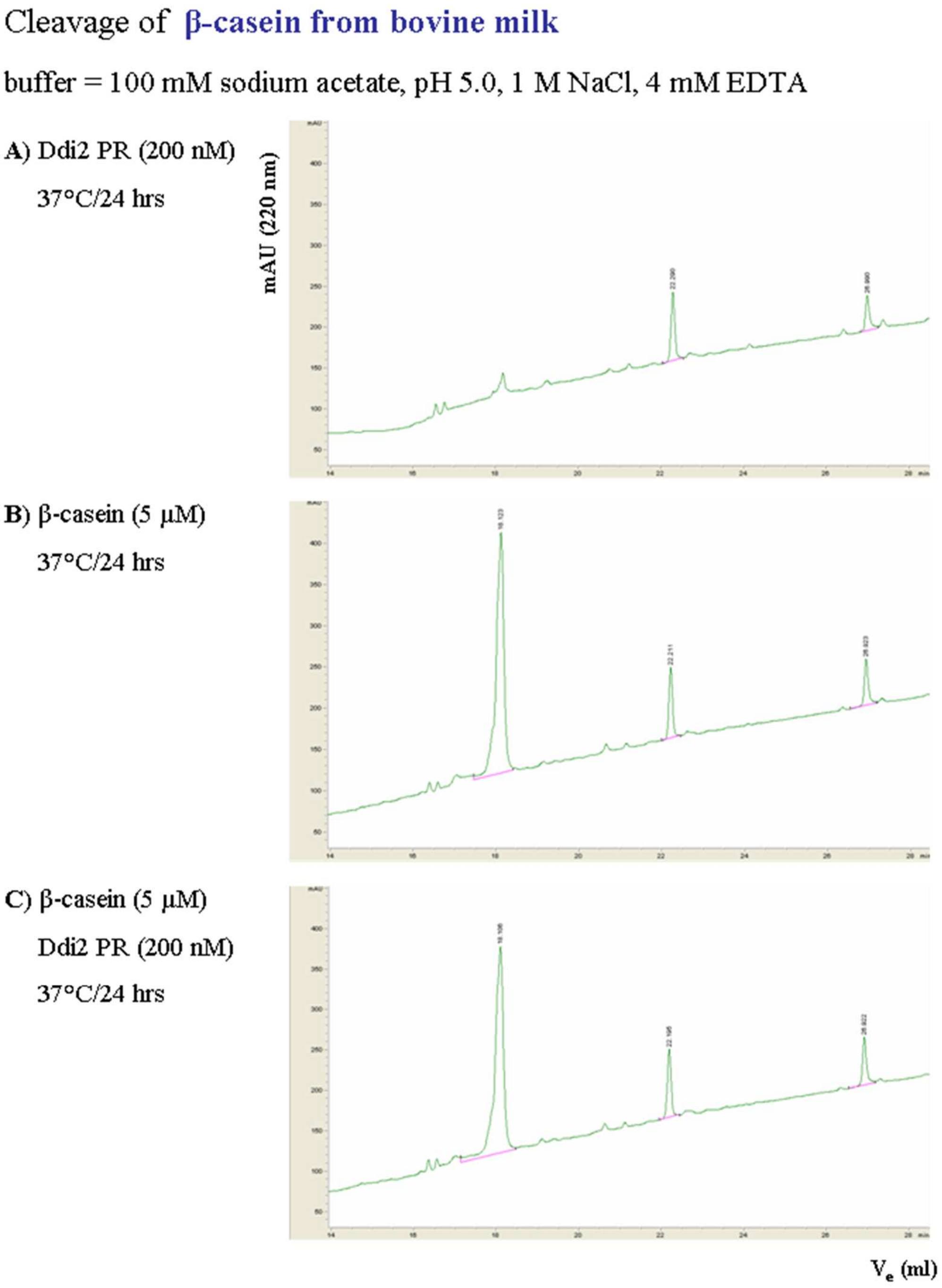

Figure S13: Testing of putative cleavage of β-casein from bovine milk by hDdi2 RVP domain at pH 5.0 with 1 M NaCl.

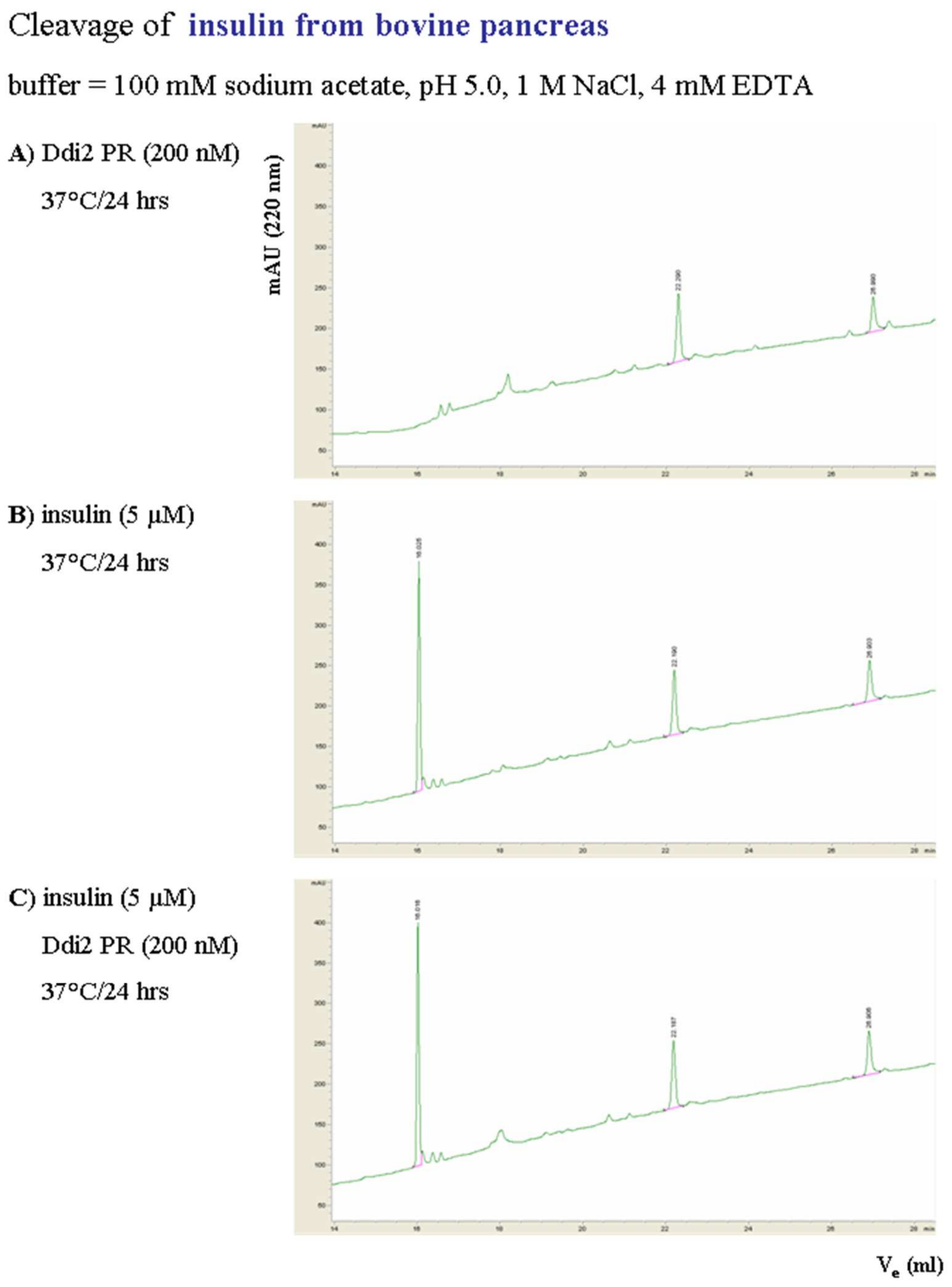

Figure S14: Testing of putative cleavage of insulin from bovine pancreas by hDdi2 RVP domain at pH 5.0 with 1 M NaCl.

Testing of putative binding of HIV protease inhibitors to the human Ddi2 RVP domain

Human Ddi2 RVP titrated with the HIV protease inhibitor darunavir[2] is used as an example.

ITC titration – Ddi2 x darunavir (DRV)

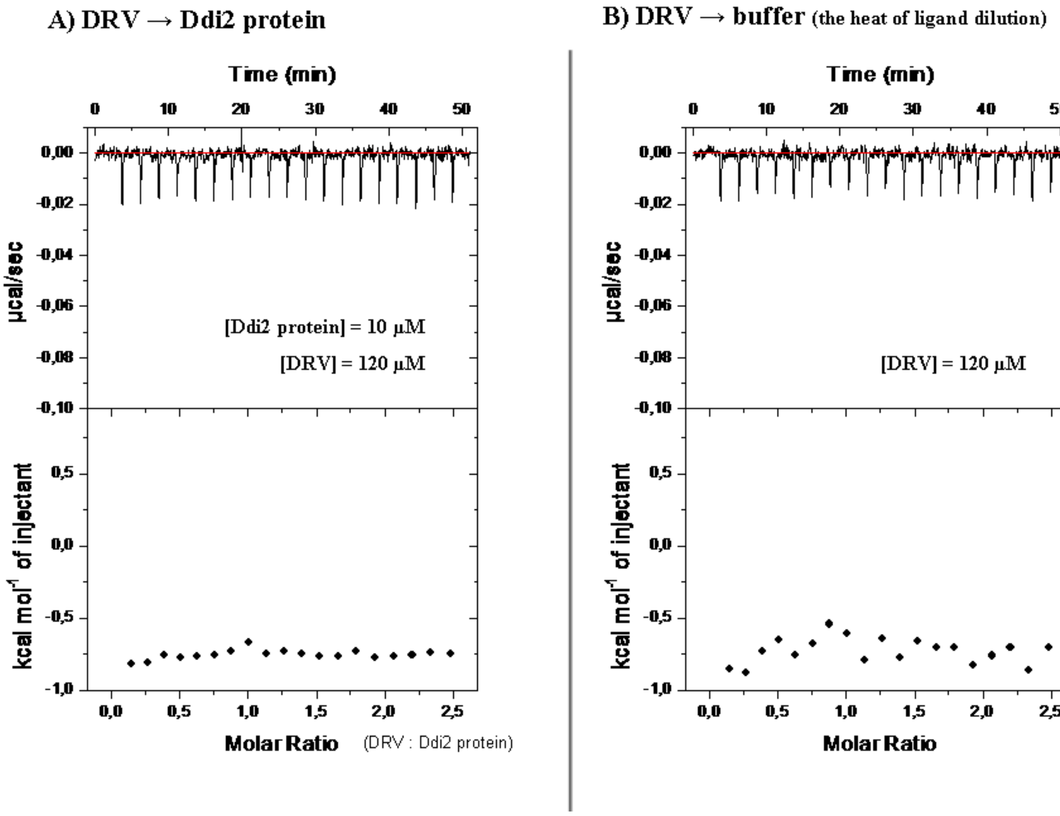

Figure S15: Testing of the putative interaction of full-length human Ddi2 with the HIV protease inhibitor darunavir. A) Calorimetric titration of darunavir with full-length hDdi2 was performed in 50 mM HEPES, pH 7.4, 150 mM NaCl at 25 °C using an Auto-iTC$_{200}$ system (MicroCal, GE Healthcare Life Sciences). Typically, 2 µl aliquots of 120 µM HIV protease inhibitor were injected stepwise into a sample cell containing 200 µl of a 10 µM protease domain of hDdi2 protein (concentration calculated to dimer; HPLC amino acid analysis was performed). The titrations were monitored by MicroCal software implemented in Origin 7.0 (MicroCal, GE Healthcare Life Sciences). B) A control dilution experiment, in which darunavir was injected into buffer alone, was also performed.

Methods

Nuclear magnetic resonance spectroscopy

A series of double and triple resonance spectra were collected for hDdi2 UBL, HDD, and RVP full-C protein constructs and UBQ. NOEs identified in NOESY, $^{15}N/^{1}H$ NOESY-HSQC, and $^{13}C/^{1}H$ HSQC-NOESY spectra were acquired with an NOE mixing time of 120 ms. All TOCSY spectra were acquired with a mixing time of 60 ms. All spectra were processed using the program Sparky (Goddard and Kneller, UCSF).

Structural calculations

The family of converged structures for hDdi2 UBL was initially calculated using Cyana 2.1[4]. NOE-derived restraints from two-dimensional NOESY and three-dimensional ^{15}N- and ^{13}C-edited NOESY spectra were used to produce preliminary structures in Cyana. Assigned chemical shifts were used to generate backbone torsion angle constraints using the program TALOS+[5]. Subsequently, five cycles of simulated annealing combined with redundant dihedral angle constraints (REDAC) were performed[6]. A group of converged structures with no notable restraint violations (distance and Van der Waals violations <0.2 Å and dihedral angle constraint violation <5°) were further refined in explicit solvent using the YASARA software with the YASARA forcefield[7]. The 40 structures for hDdi2 UBL and 30 structures for hDdi2 HDD with the lowest energy were selected. Analysis of the family of structures was carried out using the Protein Structure Validation Software suite (www.nesg.org), Molmol[8], and PyMol (PyMOL™ 0.98) (www.pymol.org).

NMR chemical shift mapping

The assignments of triple resonance spectra of hDdi2 UBL, UBQ, and hDdi2 RVP full-C construct were performed to allow specific interaction of peptides and proteins with these proteins to be monitored by changes induced in the positions of backbone signals of ^{15}N-labeled proteins in ^{15}N/^{1}H HSQC spectra. The backbone assignment for ^{15}N/^{1}H HSQC spectra of Nedd8 was derived from a published solution structure with BMRB code 10062[9]. The most significant shifts of the backbone amide groups were used for to map the binding site. The minimal shift approach was used to assess the changes in case of one-step addition of the partner[10]. Weighted-average chemical shift perturbations were defined according to the formula ($\Delta\delta = \sqrt{(\Delta\delta_H)^2 + (\Delta\delta_N * 0.2)^2}$). The titration curves were fitted with non-linear one site specific binding model in the program GraphPad Prism.

The mono UBQ - UIM peptide titration experiment was initialized by HSQC acquirement of 0.1 mM UBQ in 50 mM sodium phosphate buffer, pH 6.0, containing 3.9% DMSO. The conditions of the samples in the following titration steps remained identical with addition of UIM peptide to final concentrations of 0.69, 1.4, 2, 2.75, and 3.45 mM in individual samples. The cut-off for evaluation of the CSPs used for this experiment was 0.12. The 6 best-fitting curves corresponding to individual amino acids were used for calculation of the K_d. The control binding experiment was performed under the same conditions with 1.9 mM UIM scrambled peptide. The protein-protein interactions were monitored by acquiring ^{1}H/^{15}N HSQC spectra of free ^{15}N-labeled 0.1 mM UBQ in 50 mM sodium phosphate buffer, pH 7.4, and with 1, 2, and 5-fold molar addition of non-labeled hDdi2 RVP full-C and *vice versa*. The interaction of hDdi2 UBL with UBQ was studied by HSQC acquirement of 0.042 mM ^{15}N-labeled hDdi2 UBL with 1, 2, 4, 6, 8, and 10-fold molar addition of non-labeled bovine UBQ in 20 mM phosphate buffer, 0.5 mM DTT. The numerical cut-off for evaluation of the CSPs was 0.075. The 10 best-fitting curves corresponding to individual amino acids were used for calculation of the K_d. The reverse

experiment was carried out on 0.05 mM UBQ with 6-fold molar addition of hDdi2 ΔUIM protein construct in 50 mM phosphate buffer, 0.5 mM DTT. A control measurement was performed under the same conditions with 0.05 mM UBQ with 6-fold addition of Ddi2 HDD-RVP construct. The interactions of hDdi2 UBL with Ddi2 UIM peptide were studied by acquiring HSQC spectra of 0.05 mM protein in 50 mM sodium phosphate, pH 7.4, with addition of UIM peptide to a final concentration of 1.9 mM. A control experiment with one-step addition of scrambled peptide reaching 1.2 mM final concentration was performed. Verification of the Ddi2 UBL-UIM interaction was additionally performed on longer protein constructs by acquiring the HSQC spectra of 0.22 mM ^{15}N-labeled hDdi2 FL protein and HSQC spectra of 0.093 mM hDdi2 ΔUIM in 50 mM sodium phosphate buffer, pH 7.4, with 0.1 mM DTT. Other potential intramolecular interactions and UBL domain flexibility were characterized by superimposition of the HSQC spectra of full-length hDdi2 protein and HSQC spectra of 0.05 mM hDdi2 UBL domain. Nedd8 interaction with the two peptides was verified via an identical acquisition with 0.03 mM protein under two different conditions: 50 mM sodium acetate, pH 5, and 50 mM sodium phosphate, pH 7. The final addition of the UIM peptide at pH 5 was up to 1.2 mM. At pH 7, the final concentrations of the UIM peptide and the scrambled version were 1.9 and 1.2 mM, respectively. The interaction of hDdi2 UBL with Nedd8 was studied by 6-fold molar addition of Nedd8 to 0.05 mM UBL in 50 mM sodium phosphate buffer, pH 7.4, 0.5 mM DTT.

Table S1: NMR constraints and structural statistics for hDdi2 UBL and hDdi2 HDD

	**hDdi2 UBL**		**hDdi2 HDD**	
**NMR distance & dihedral constraints**				
Distance constraints				
Total NOE	1095		1894	
Intra-residue	287		487	
Inter-residue	808		1407	
Sequential (|i-j| = 1)	291		522	
Medium-range (|i-j| < 4)	170		593	
Long-range (|i-j > 5)	347		292	
Total dihedral angle restraints	132		108	
phi	66		54	
psi	66		54	
**Structure statistics**				
Violations (mean and s.d.)				
Max. dihedral angle violation (º)	5.00		5.10	
Max. distance constraint violation (Å)	0.35		0.49	
Deviations from idealized geometry				
Bond lengths (Å)	0.011		0.012	
Bond angles (º)	1.4		1.5	
Ramachandran plot summary				
Most favoured regions	91.2%		93.9%	
Additionally allowed regions	8.6%		6.0%	
Generously allowed regions	0.2%		0.1%	
Disallowed regions	0.0%		0.0%	
Average pairwise r.m.s.d. foe[*] (Å)	*Ordered*[a]	*all residues*	*Orderea*[b]	*all residues*
Heavy	1.0	4.8	4.8	8.1
Backbone	0.4	4.3	4.5	7.8

[*] 40 hDdi2 UBL and 30 hDdi2 HDD structures were used in r.m.s.d. calculations

[a] ordered residue range – residues with defined dihedral angle order parameters. S(phi)+S(psi)>=1.8

Table S2: X-ray diffraction data processing and refinement statistics for hDdi2 212-360.

Data collection	
Wavelength (Å)	0.91573
Space group	$P2_12_12$
Unit cell dimensions (Å)	66.87 86.40 52.12
(°)	90.0 90.0 90.0
Mosaicity (°)	0.9
Images	235
Oscillation angle (°)	0.5
Resolution (Å)	26.7 – 1.9 (2.0 – 1.9)
Unique reflections	21198 (2439)
Completeness (%)	87.2 (69.7) *
Multiplicity	4.6 (4.7)
R_{meas}[a]<br><I>/<σ(I)>	0.113 (0.422)<br>9.5 (3.6)
Wilson B ($Å^2$)[b]	27.6
Refinement	
Resolution (Å)	33.7 – 1.9 (1.94 -1.90)
R_{work} (%)	20.8 (39.5)
R_{free} (%)	25.6 (45.7)
Rms deviations	
Bond length (Å)	0.014
Bond angle (°)	1.75
Protein atoms	2033
Solvent atoms	86
Average B-factor[b]	26.0
Most/additional favoured regions f-y space (%)	99.2/1.2
Coordinate error (Å) [c]	0.17/0.11 (based on R value/Maxim Likelihood)

[a] R_{meas} defined in ref.[11]. [b] Wilson B by Sfcheck program[12] from CCP4 suite[13]. [c] Calculated by Refmac5[14].

* Ice ring areas were excluded from integration.

References

1 Walters, K. J., Lech, P. J., Goh, A. M., Wang, Q. H. & Howley, P. M. DNA-repair protein hHR23a alters its protein structure upon binding proteasomal subunit S5a. *P Natl Acad Sci USA* **100**, 12694-12699, (2003).

2 Koh, Y. *et al.* Novel bis-tetrahydrofuranylurethane-containin nonpeptidic protease inhibitor (PI) UIC-94017 (TMC114) with potent activity against multi-PI-resistant human immunodeficiency virus in vitro. *Antimicrob Agents Ch* **47**, 3123-3129, (2003).

3 Cornilescu, G., Marquardt, J. L., Ottiger, M. & Bax, A. Validation of protein structure from anisotropic carbonyl chemical shifts in a dilute liquid crystalline phase. *J Am Chem Soc* **120**, 6836-6837, (1998).

4 Herrmann, T., Guntert, P. & Wuthrich, K. Protein NMR structure determination with automated NOE-identification in the NOESY spectra using the new software ATNOS. *J Biomol Nmr* **24**, 171-189, (2002).

5 Shen, Y., Delaglio, F., Cornilescu, G. & Bax, A. TALOS plus : a hybrid method for predicting protein backbone torsion angles from NMR chemical shifts. *J Biomol Nmr* **44**, 213-223, (2009).

6 Guntert, P. *et al.* Structure Determination of the Antp(C39-]S) Homeodomain from Nuclear-Magnetic-Resonance Data in Solution Using a Novel Strategy for the Structure Calculation with the Programs Diana, Caliba, Habas and Glomsa. *Journal of Molecular Biology* **217**, 531-540, (1991).

7 Harjes, E. *et al.* GTP-Ras disrupts the intramolecular complex of C1 and RA domains of Nore1. *Structure* **14**, 881-888, (2006).

8 Koradi, R., Billeter, M. & Wuthrich, K. MOLMOL: A program for display and analysis of macromolecular structures. *J Mol Graphics* **14**, 51-&, (1996).

9 Sakata, E. *et al.* Direct interactions between NEDD8 and ubiquitin E2 conjugating enzymes upregulate cullin-based E3 ligase activity. *Nat Struct Mol Biol* **14**, 167-168, (2007).

10 Farmer, B. T. Localizing the NADP(+) binding site on the MurB enzyme by NMR. *Nat Struct Biol* **3**, 995-997, (1996).

11 Diederichs, K. & Karplus, P. A. Improved R-factors for diffraction data analysis in macromolecular crystallography. *Nat Struct Biol* **4**, 269-275, (1997).

12 Vaguine, A. A., Richelle, J. & Wodak, S. J. SFCHECK: a unified set of procedures for evaluating the quality of macromolecular structure-factor data and their agreement with the atomic model. *Acta Crystallogr D* **55**, 191-205, (1999).

13 Winn, M. D. *et al.* Overview of the CCP4 suite and current developments. *Acta Crystallogr D* **67**, 235-242, (2011).

14 Murshudov, G. N., Vagin, A. A. & Dodson, E. J. Refinement of macromolecular structures by the maximum-likelihood method. *Acta Crystallogr D* **53**, 240-255, (1997).
